# Circuit variability interacts with excitatory-inhibitory diversity of interneurons to regulate network encoding capacity

**DOI:** 10.1038/s41598-018-26286-8

**Published:** 2018-05-23

**Authors:** Kuo-Ting Tsai, Chin-Kun Hu, Kuan-Wei Li, Wen-Liang Hwang, Ya-Hui Chou

**Affiliations:** 1 Institute of Cellular and Organismic Biology, Academia Sinica, Taipei, Taiwan; 2 Institute of Physics, Academia Sinica, Taipei, Taiwan; 3 Neuroscience Program of Academia Sinica, Academia Sinica, Taipei, Taiwan; 4 National Center for Theoretical Sciences, National Tsing Hua University, Hsinchu, Taiwan; 5 Business School, University of Shanghai for Science and Technology, Shanghai, China; 6 Institute of Information Science, Academia Sinica, Taipei, Taiwan

## Abstract

Local interneurons (LNs) in the *Drosophila* olfactory system exhibit neuronal diversity and variability, yet it is still unknown how these features impact information encoding capacity and reliability in a complex LN network. We employed two strategies to construct a diverse excitatory-inhibitory neural network beginning with a ring network structure and then introduced distinct types of inhibitory interneurons and circuit variability to the simulated network. The continuity of activity within the node ensemble (oscillation pattern) was used as a readout to describe the temporal dynamics of network activity. We found that inhibitory interneurons enhance the encoding capacity by protecting the network from extremely short activation periods when the network wiring complexity is very high. In addition, distinct types of interneurons have differential effects on encoding capacity and reliability. Circuit variability may enhance the encoding reliability, with or without compromising encoding capacity. Therefore, we have described how circuit variability of interneurons may interact with excitatory-inhibitory diversity to enhance the encoding capacity and distinguishability of neural networks. In this work, we evaluate the effects of different types and degrees of connection diversity on a ring model, which may simulate interneuron networks in the *Drosophila* olfactory system or other biological systems.

## Introduction

Animals sense environmental stimuli and initiate appropriate behavioral responses through the action of neural circuits. In the complex olfactory circuit, the number of olfactory receptors (ORs) in many species is far fewer than the environmental stimuli that the species can detect^1^, and the organism uses a limited number of ORs and olfactory receptor neurons (ORNs) to distinguish and encode target information from hundreds or thousands of background stimuli in a dynamic environment. Thus, the sensory circuit in the olfactory bulb, the first olfactory information processing center in mammals, should be able to reconcile possible trade-offs between encoding reliability and encoding capacity, while possessing a certain degree of coding flexibility^1–3^.

**Figure 1:**
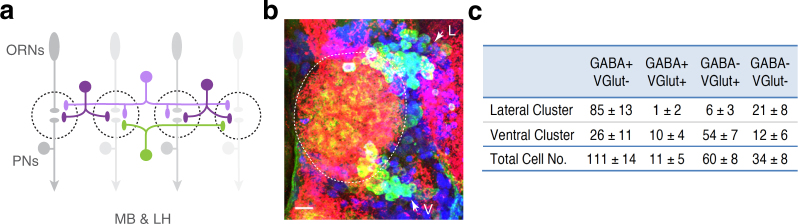
*Drosophila* LN network exhibits high ratio of inhibitory to excitatory neurons. (**a**) Schematic of the *Drosophila* olfactory system. Dashed circles denote individual glomeruli in the AL, where axons of ORN (gray ovals), dendrites of PNs (gray circles), and the processes of excitatory LNs (green) and inhibitory LNs (magenta) form extensive synaptic connections. Different shades of green and magenta represent different types of corresponding neurons. MB, mushroom body. LH, lateral horn. (**b**) Adult fly brains were stained with anti-GABA (blue), anti-VGlut (red), and anti-mCD8 (green) antibodies to visualize GABAergic, glutamatergic and almost all LNs, respectively. White dashed line contours the AL. Arrows indicate lateral (L) and ventral (V) LN clusters. Scale bar, 10 μm. (**c**) The table shows estimated numbers of GABA- and/or VGlut-expression LNs within the AL (mean ± SD, n = 5). LNs expressing either GABA, VGlut or both are inhibitory LNs; GABA-negative and VGlut-negative LNs are excitatory LNs.

Compared to mammalian systems, the *Drosophila* olfactory system exhibits similar, but much simpler, circuit wiring^4^ (Fig. 1a). In *Drosophila*, odorants are represented non-linearly in input terminals and cognate output terminals, suggesting that signals passed to sensory neuron termini are modulated by interneurons in the input channels^5,6^. Such modulation occurs mainly through feedback inhibition from inhibitory local interneurons (LNs) to ORNs – a process known as gain control^7,8^ – or lateral inhibition and lateral excitation from LNs to projection neurons (PNs) or other LNs^9–14^. In addition, network flexibility has been recently demonstrated in the antennal lobe (AL)^10,15–20^, and it is not unique to the fly olfactory circuit. Indeed, mitral cells (the vertebrate analog of fly PNs) exhibit intrinsic heterogeneity, which decorrelates neuronal firing and increases coding capacity^18^. Intuitively, these regulatory processes should result in pattern decorrelation^2^, but collective variations in subsets of neurons within a circuit have been shown to produce consistent network activity, thus maintaining encoding reliability^21^. However, it is still an open question whether any trade-off exists between coding capacity (pattern decorrelation) and reliability in early olfactory information processing.

Our previous work demonstrated that *Drosophila* olfactory LNs are highly diverse and variable, both in morphology and electrical properties^15^. Yet it is not clear why the circuit needs highly diverse LNs, or how LN variability may affect the encoding capacity. Few studies have considered the effects of interneuron diversity on network activity^22–27^, while much effort has been devoted to understanding the effects of heterogeneity on firing threshold value^28–30^ or intrinsic biophysical properties^31,32^. As such, heterogeneity has been found to largely affect firing rates and/or signal representation in the network^28,32^. Therefore, we focused our study on describing the effects of morphological diversity on network activity.

LNs, ORNs and PNs form multiple feedback and feed-forward loops, and the majority of previous simulation work has been performed using networks that consist of ORNs, LNs and/or PNs. However, if we want to understand how LN network activity changes temporally and spatially, it is difficult to simultaneously consider time-dependent inputs from ORNs and PNs. Therefore, we began by using a ring model as a basic representation of an oscillating isolated LN network (interactions with ORNs and PNs were excluded). With this model, we asked (1) why biological networks might exhibit high levels of diversity, (2) if morphological diversity contributes to the encoding capacity of a network consisting of inhibitory and excitatory neurons, and (3) if so, whether and how circuit variability drives the circuit toward a balance between encoding capacity and reliability.

To address these questions, we constructed networks consisting of excitatory and inhibitory interneurons, and introduced distinct types of variability to the simulated networks. Our aims were to understand to what extent the transmission of neural firing could be modeled in these neural ensembles, and to develop a way to better understand the spatial and temporal dynamics of neural firing patterns. The continuity of activity within the node ensemble (oscillation pattern) was used as a readout to describe the temporal dynamics of network activity. Our work may explain why LN diversity is required for the *Drosophila* olfactory circuit. In addition, our findings describe how circuit variability of LNs may combine with inhibition to enhance both the encoding capacity and distinguishability of LN networks. Such mechanisms are likely to be generalizable to interneuron networks in other circuits and other species.

## Results

### The ratio of excitatory and inhibitory LNs is approximately 1:5.4 in the AL

Around the *Drosophila* AL, LNs are restricted to the lateral and ventral clusters, each containing ~100 LNs^15^ (Fig. 1b). In addition to excitatory cholinergic LNs and inhibitory GABAergic LNs, glutamatergic LNs were recently discovered to be inhibitory^15,33^. In this study, we first determined the ratio of excitatory LNs and inhibitory LNs in the AL. We co-stained γ-aminobutyric acid (GABA) and Vesicular glutamate transporter (VGlut) to simultaneously visualize GABAergic and glutamatergic LNs. Thus, the entire population of inhibitory LNs was labeled in each brain (Fig. 1b). Quantification showed the ratio of excitatory to inhibitory LNs is approximately 1:5.4 (Fig. 1c).

### Constructing the basic regular interneuron network

**Figure 2:**
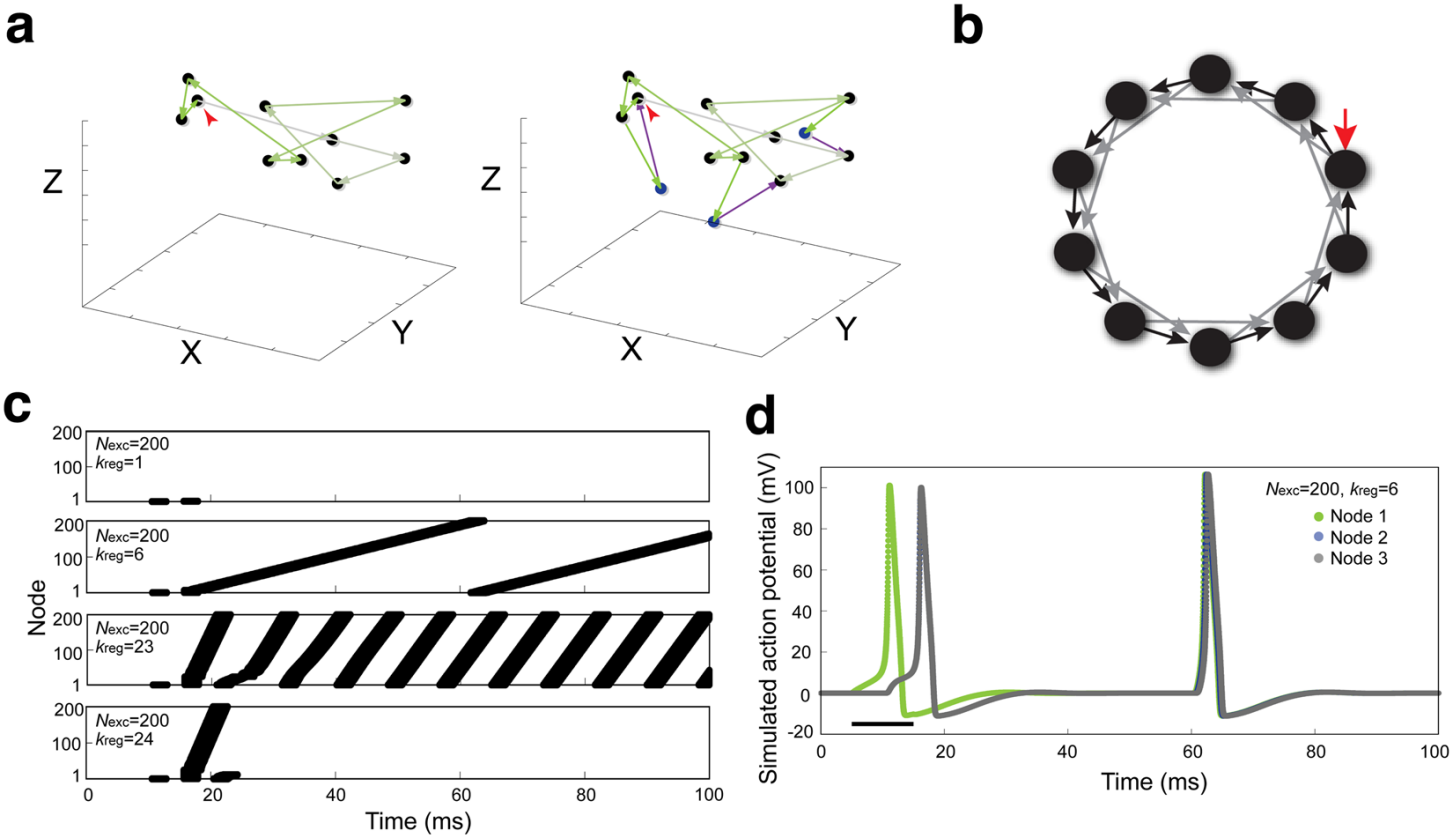
Building a regular circular network for simulating LN network. (**a**) Conceptual illustration of the spatial and temporal dynamics of neuronal activity in an ensemble. Neurons are randomly plotted in a representation of a three-dimensional space. *Left*: a closed loop network with solely excitatory neurons (black dots). *Right*: a closed loop network with excitatory (black dots) and inhibitory (blue dots) neurons. The activation flow is demonstrated by green (excitation) or magenta (inhibition) arrows. The darker green arrows represent later events. Red arrows indicate the initial stimulus. (**b**) Diagram of a regular circular network. Nodes (filled circles) represent individual LNs. Synapses from one neuron to the closest and second closest neurons are indicated by black and gray arrows (edges), respectively. The red arrow is a sole stimulus injected to initiate firing. Simulated networks included 200 excitatory nodes (*N*_exc_) and varying numbers of regular activation edges (*k*_reg_). For simplicity, only 10 excitatory nodes (*N*_exc_) and 2 regular activation edges (*k*_reg_) of each node are shown. (**c**) *k*_reg_ was varied in a network with *N*_exc_ = 200. The dynamics of network activation are shown as spike raster plots, with the vertical axis denoting the identity of each node in the network (the node receiving injected stimulus is assigned as node 1) and the horizontal axis denoting simulation time. (**d**) The activation dynamics of individual nodes (node 1, green; node 2, blue; node 3, gray) are shown as simulated action potentials over time in a regular network (*N*_exc_ = 200, *k*_reg_ = 6). Only the first three nodes are shown. The black bar indicates the period of stimulation. Node 2 and node 3 are simultaneously activated by node 1. Consequently, the action potential of node 2 overlaps with node 3 and the two activations are not clearly visible in the figure.

We began our simulations with a regular ring network and progressively increased the complexity by diversifying connections. One may imagine that in a three-dimensional space, the most basic version of a neural circuit could be excitatory neurons within a neural ensemble that form regular connections and fire sequentially (Left, Fig. 2a). This network will easily produce oscillations that are observed in biological systems. As more complexity (i.e. inhibitory connections and irregular connectivity) is introduced, more irregularities in firing sequence and heterogeneous activity may occur (Right, Fig. 2a). Using this approach, we can begin to understand the effects of morphological diversity and wiring variability on spatial and temporal dynamics of neural firing patterns.

Herein, the term ‘network’ refers to a particular network topology (e.g. *N*_exc_ = 20, *k*_reg_ = 2), while ‘network structure’ refers to 5000 simulated results generated from a given network. To determine how inhibition and circuit variability of LNs affects the dynamics of global neural activity in the AL, we first conceived a simplified network that solely consists of excitatory LNs (i.e. lacking PNs, ORNs and inhibitory LNs; Supplementary Fig. S1a). To simulate this network, we constructed a regular circular network structure (Fig. 2b). Each node represents an excitatory LN, which connects to and activates to the closest and second closest neuron through two directed edges (*k*_reg_ = 2). Accordingly, each neuron also receives excitatory inputs from two others.

We then modified the Hodgkin-Huxley model for neurons^34^ by dividing applied current *I* into two terms, a stimulating current *I*_sti_ and a synaptic current *I*_syn_, to enable integration of excitatory and inhibitory information in a node for later simulation (see Model and Table S1 for the parameters). Because this network structure is regular and symmetric, a single stimulus (red arrow in Fig. 2b) induced orderly firing of all the nodes (Fig. 2c). Previous electrophysiological experiments have demonstrated that LN ensembles are activated over a time period during and/or after odor stimulations^6,15^, so we used the continuity of activity within the ensemble as a readout to evaluate the effects of two basic parameters, the number of nodes (*N*_exc_) and the number of edges (*k*_reg_) (Supplementary Fig. S2). When *N*_exc_ was fixed at 200 and *k*_reg_ varied, we observed three types of firing patterns (Fig. 2c). When *k*_reg_ was 1, stimulation occurred sequentially, however, a single stimulation was not sufficient to activate all nodes, and dissipative transmission caused rapid extinction of activity in the ensemble. As *k*_reg_ increased, the first activated node was sufficient to activate the next two and so on, creating a chain reaction-like activation pattern (*k*_reg_ = 6 or 23). When *k*_reg_ was high (*k*_reg_ = 24), network activation was initiated but died out soon after. This is likely because when *k*_reg_ is large, signals are transmitted very fast. As a consequence, when the signal is transmitted back to the first node in the second cycle, the node is still hyperpolarized, preventing reactivation. Regular networks with *N*_exc_ = 20 or 100 exhibited similar types of firing patterns when *k*_reg_ increased (Supplementary Fig. S2a,b). We further examined the firing patterns of individual nodes in the second type of activation (*N*_exc_ = 200, *k*_reg_ = 6). As expected, the injected stimulus induced Node 1 firing, followed by simultaneous firing of Nodes 2 and 3 in the first activation cycle. From the second cycle onwards, the firing pattern of each node developed a regular oscillation pattern, with a certain firing delay time between each node (Fig. 2d). We hereafter describe the continuous activity of individual nodes in a given network structure as the ‘network activity’ and describe the dynamic network activity of all nodes through time as the ‘oscillation pattern’ or ‘activation pattern’ (see below).

### Randomly switching excitatory nodes to inhibitory nodes to simulate interneuron networks

**Figure 3:**
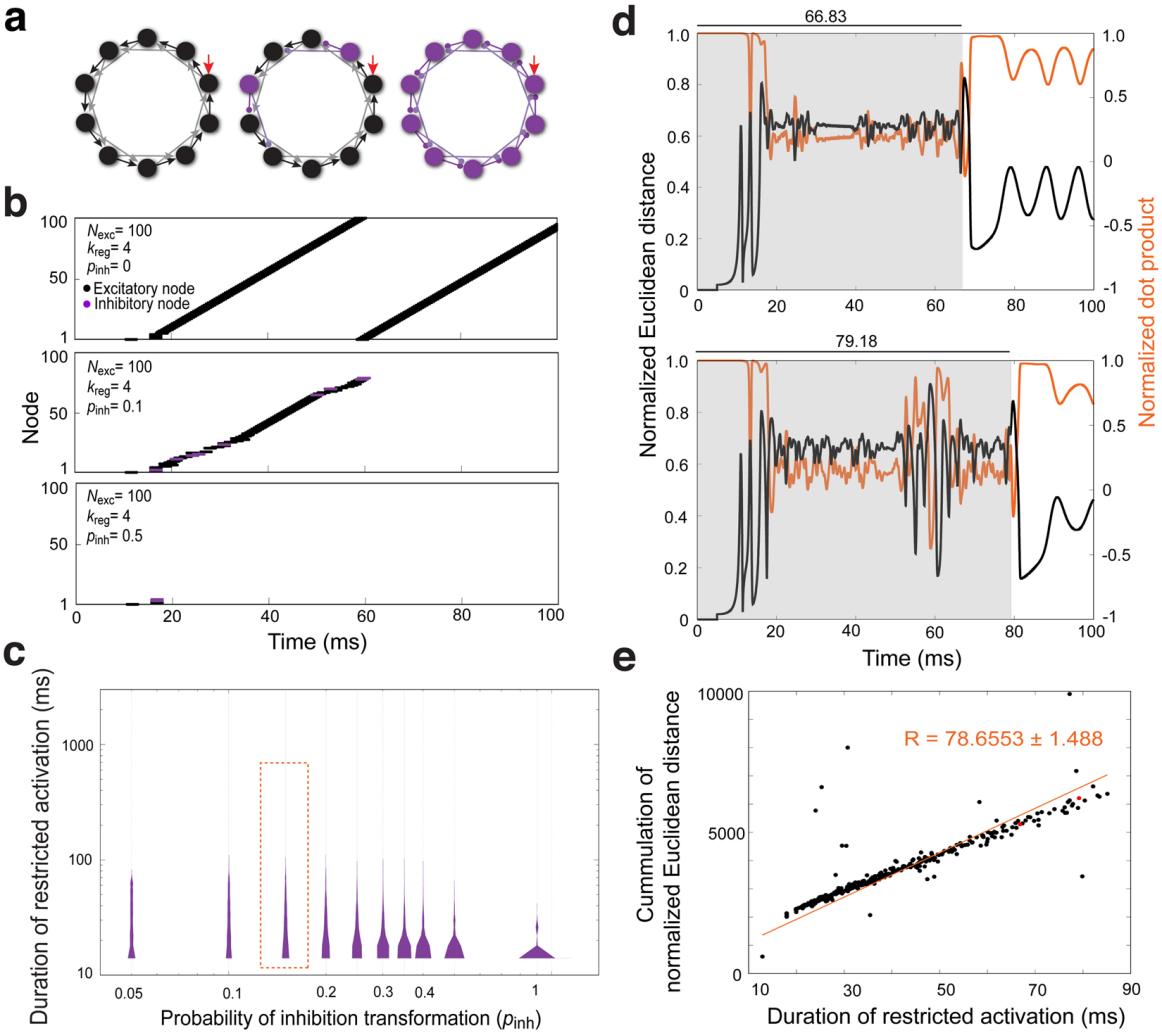
Random assignment of inhibitory nodes to construct an excitatory-inhibitory network. (**a**) Diagram depicting random assignment of excitatory and inhibitory nodes in the regular network. Ten nodes and 2 directed edges are shown. *Left*: *p*_inh_ = 0. *Middle*: inhibitory nodes and edges are colored magenta. As the probability of inhibitory transformation (*p*_inh_) increases, more nodes become inhibitory. *Right*: *p*_inh_ = 1. (**b**) The dynamics of network (*N*_exc_ = 100, *k*_reg_ = 4) activation are shown as spike raster plots. Inhibitory nodes are colored magenta. *Top*: At *p*_inh_ = 0, ceaseless activation is observed. *Middle*: When *p*_inh_ = 0.1, some network structures show restricted activation with slightly irregular activation patterns. *Bottom*: When *p*_inh_ = 0.5, most networks exhibit restricted activation (example trace, 17.97 ms). (**c**) Five thousand networks (*N*_exc_ = 100, *k*_reg_ = 4) were simulated by randomly switching nodes from an excitatory to inhibitory at a given *p*_inh_. The durations of restricted activation (magenta, ∈ [15,1000]) are shown as violin plots. When *p*_inh_ = 0, activation was not restricted (data not shown). A statistically significant difference was found among groups by Kruskal-Wallis *H*-test (*p* < 0.0001) (Table S2). (**d**,**e**) Longer network activation durations may reflect greater distinguishability. (**d**) The membrane potentials of all nodes in a given simulated network structure (*N*_exc_ = 100, *k*_reg_ = 4, *p*_inh_ = 0.15) were compiled as a vector. Two stimulating currents were separately applied to the same network structure. Normalized Euclidean distance (black) and normalized dot product (orange) for the resulting vectors were used to quantify distinguishability. Only the first 100 ms are shown. *Top panel*: A single network structure with activation durations of 66.83 and 68.58 ms from *I*_*i*,sti_ = 2.5 and 2.4, respectively. The gray zone indicates time during which both network structures were activated. *Bottom panel:* Another network structure for which activation durations of 79.18 ms and 81.05 ms were observed from *I*_*i*,sti_ = 2.5 and 2.4, respectively. (**e**) Cumulative Euclidean distance was derived from 500 simulated network structures in (**c**) (outlined by orange dashed line). The two network structures shown in (**d**) are plotted in red. The cumulative normalized Euclidean distance is proportional to the duration of restricted activation, suggesting the later reflects the distinguishability of networks. R, slop of linear fitting.

In the *Drosophila* AL, the relative connection density of excitatory and inhibitory LNs is undescribed, as are the connections between LNs^15^. Therefore, we began with a basic regular network structure and randomly switched the excitatory nodes to inhibitory nodes, with a probability of inhibitory transformation *p*_inh_ ∈ [0, 1] (Fig. 3a), to build networks with both excitatory and inhibitory LNs (Supplementary Fig. S1b). When *p*_inh_ = 0, all nodes are excitatory (same as Fig. 2b). If *p*_inh_ = 1 (Fig. 3a, right), all nodes are inhibitory.

Conceptually, a basic regular network with *N*_exc_ = 200 would be suitable for experimentation because it represents the number of LNs in the AL. However, we found the basic regulatory networks with *N*_exc_ = 100 showed similar firing patterns to networks with *N*_exc_ = 200 (Supplementary Fig. S2). To make the simulation less computationally taxing, we set the basic regular network structure to *N*_exc_ = 100 and *k*_reg_ = 4. When the probability of inhibitory transformation *p*_inh_ was applied to a regular network, any of the original excitatory nodes could be switched to an inhibitory node. When excitatory nodes are switched to inhibitory, those nodes inhibited the nodes that are immediately downstream. Consequently, the chain reaction-like network activation was broken, producing diverse network activation patterns (Fig. 3b, middle panel). When *p*_inh_ was high (~0.5), the network activity was terminated after a short activation period (Fig. 3b, bottom panel). When network activity ceased during the simulation period, the network structure was considered to have undergone activation restriction (Supplementary Fig. 3a, Supplementary note 1). In further simulation work, we used the duration of restricted activation as our main endpoint.

Information theory, such as Shannon entropy, can describe the amount of information in a system. Such theories clearly address the amount of information that may be encoded by the network structures; however, they cannot describe the dynamics of information encoding over time. To better examine the structural and temporal dynamics of information in the simulated neural networks, we used collective activity oscillation patterns of all simulated network structures derived from a given network to describe network information. In the simulated network structure, a single stimulation may trigger sequential firing of individual nodes and thus lead to cycling signal transmission among nodes. This cycling appeared as oscillating patterns that may continue for a period of time, and may differ from trial to trial (Figs 2a and 3b). Therefore, the collective oscillation patterns of all nodes – but not individual nodes – represent the global activity dynamics of a given network structure. As such, network oscillation patterns describe the structural and temporal features of a neuronal population (Fig. 2a).

We examined the activation durations of networks with restricted activation under different values of *p*_inh_ (Fig. 3c). When *p*_inh_ was large enough (e.g. *p*_inh_ = 0.3), the duration of restricted activation for most networks was drastically reduced because the networks endured strong inhibition. We envision that longer durations of restricted activation allow for greater numbers of distinct temporal activation patterns. In other words, longer activation durations allow more information to be encoded. In this way, temporal dynamics would directly reflect encoding capacity. However, it is also possible that for any given duration, the network structures will exhibit similar activation patterns, reducing the time dependence of encoding capacity.

To test the idea that longer durations of network activation may confer better distinguishability, we analyzed our simulation data by two methods, Euclidean distance and dot products. Over the time-course of the simulation, the firing potential of all nodes in a network structure was treated as a vector. Two different stimulating currents (*I*_*i*,sti_ = 2.5 and 2.4 in Fig. 3d) were separately applied to the same network structure (*N*_exc_ = 100, *k*_reg_ = 4, *p*_inh_ = 0.15 in Fig. 3d), and the two vectors were compared by calculating Euclidean distance and dot products over the simulation time. We found that when activation lasted longer, the normalized Euclidean distance remained larger than 0, and the corresponding normalized inner product remained smaller than 1 (Fig. 3d). These results indicated longer durations of activation allowed the network structure to distinguish between two similar stimuli with increasing clarity. Plotting the cumulation of normalized Euclidean distance and restricted activation duration of 500 simulated network structures of the same network (outlined by dashed rectangle, Fig. 3c) further supported this idea (Fig. 3e). Therefore, the duration of network activation can be interpreted as the encoding capacity of a network. In addition, when a network structure generates a broad distribution of network activation durations, the activation duration may be more variable from trial to trial, conferring lower encoding reliability. For instance, when comparing network structures with *p*_inh_ = 0.05 and *p*_inh_ = 0.5, the structure with *p*_inh_ = 0.5 has less encoding capacity but higher encoding reliability.

Our results suggest that including some inhibitory nodes lengthens the duration of network activation, conferring greater encoding capacity. However, very strong inhibition may diminish encoding capacity. The ratio of excitatory to inhibitory LNs is 1:5.4 in the biological olfactory LN network, which would correspond to a simulated network with *p*_inh_ = 0.84. Under this condition, the duration of restricted network activation was extremely short, suggesting that encoding capacity would be suboptimal and additional factors are involved in LN network activation. This inspired us to investigate whether morphological variability could be one such factor.

**Figure 4:**
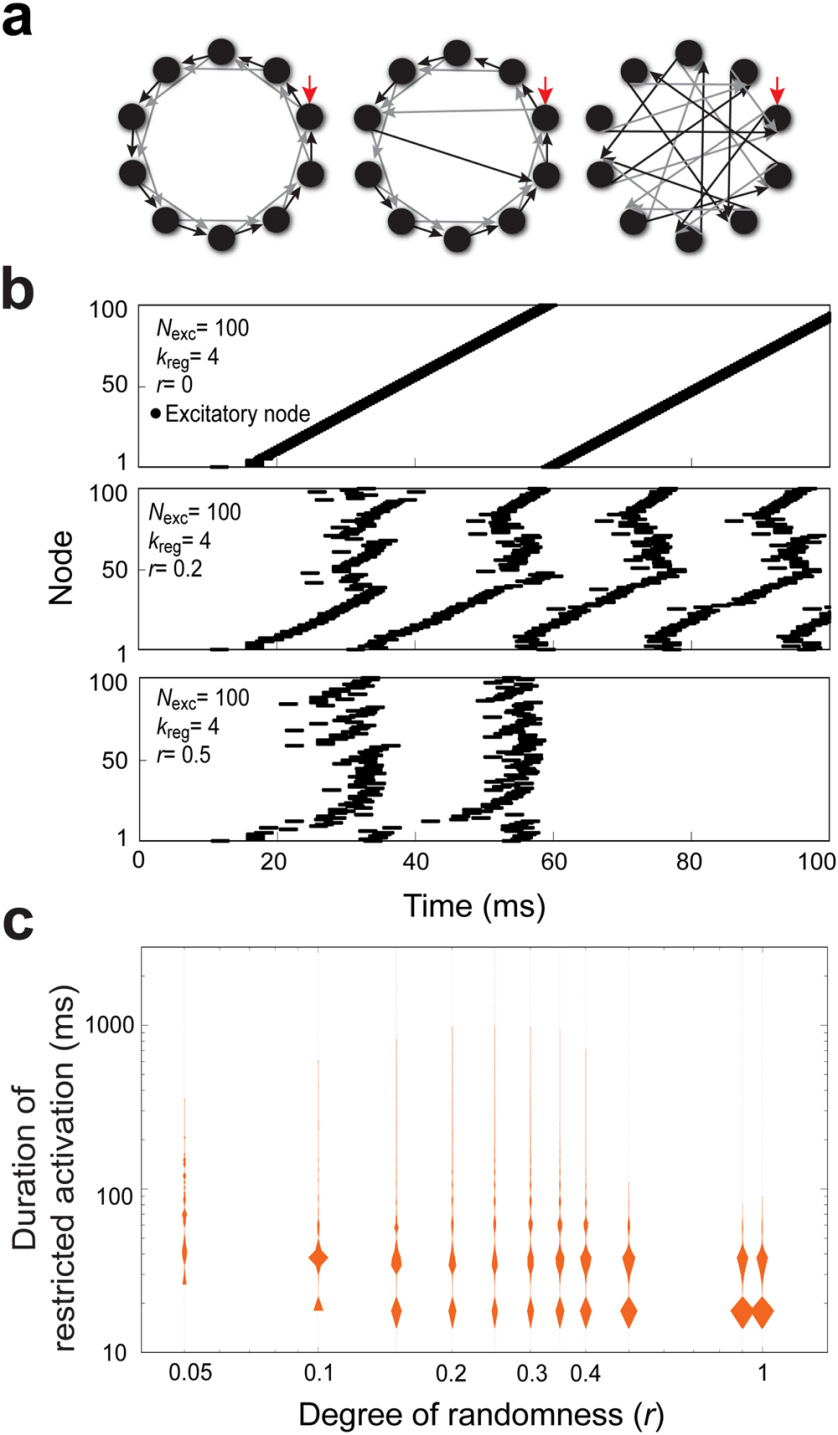
Random rewiring of node connections in a regular network to simulate LN variability. (**a**) Diagram depicting random rewiring of the connections between nodes in the basic regular network. *Left*: the basic regular network structure, in which the degree of randomness (*r*) is 0. *Middle*: network structure after one of the two directed edges from two individual nodes was rewired. The larger the degree of randomness, the more frequently directed edges are rewired. *Right*: when the degree of randomness of the network structure is 1, both directed edges from all individual nodes are rewired. (**b**) The dynamics of network (*N*_exc_ = 100, *k*_reg_ = 4) activation are shown as spike raster plots. *Top*: When *r* = 0, the networks show ceaseless and regular activation patterns. *Middle*: When *r* = 0.2, some simulated networks showed irregular restricted activation patterns (example trace, 458.85 ms). *Bottom*: With a higher degree of randomness, *r* = 0.5, some simulated networks showed irregular and restricted activation patterns, with short duration of activation (example trace, 58.8 ms). (**c**) Five thousand network structures were simulated by randomly rewiring the directed edges from nodes in the basic regular network (*N*_exc_ = 100, *k*_reg_ = 4) at a given *r*. When *r* = 0, none of the simulated network structures exhibited restricted activation, and therefore, these data are not shown on the plot. The duration of restricted activation (orange, ∈ [15,1000]) is shown as violin plots. A statistically significant difference among groups was found by the Kruskal-Wallis *H*-test (*p* < 0.0001) (Table S2).

We examined whether irregular connections may influence the global activation of the network. The easiest way to introduce irregularity to regular networks is by rearranging directed edges among nodes^35^. We therefore modified a regular network structure with solely excitatory nodes (*N*_exc_ = 100, *k*_reg_ = 4) by randomly rewiring the connections between nodes. Rewiring proceeded according to a degree of randomness *r* ∈ [0, 1], which dictated whether a given node is rewired or not. This allowed us to test the effect of random connections between interneurons on network activity (Supplementary Fig. S1c and see below). The four randomly rewired edges from a given excitatory node were allowed to connect to any other nodes. As *r* increases, more directed edges are rewired (middle, Fig. 4a). The number of possible network structures can be as many as ${[\frac{({N}_{{\rm{exc}}}+{k}_{{\rm{reg}}}-2)!}{{k}_{{\rm{reg}}}!({N}_{{\rm{exc}}}-2)!}]}^{{N}_{{\rm{exc}}}}$ for *r* = 1, in which all directed edges are rewired, and the network connectivity is completely random (right, Fig. 4a). As expected, the oscillation patterns of network activity were more irregular when random wiring was introduced to the network (Fig. 4b). As the degree of introduced randomness increased, the network activity exhibited restriction (Fig. 4b, bottom, Supplementary Fig. S3b).

To better understand how the degree of randomness may influence network oscillation patterns, we analyzed the duration of restricted activation according to randomness (Fig. 4c). When a low degree of randomness (e.g. *r* = 0.05–0.3) was introduced to the network structure, the distribution of restricted activation durations was multimodal. Interestingly, when *r* reached a large enough value (e.g. *r* > 0.4), the distribution of activation durations was characterized by shorter durations and a clear bimodal distribution. We envision that highly similar activation durations represents better encoding reliability than a broad distribution. This effect of randomness on activation duration demonstrates that introducing a low degree of randomness enhances the encoding capacity, while a high degree of randomness decreases encoding capacity and increases reliability.

### Randomly rewiring the connections of existing excitatory and inhibitory nodes enhances the encoding capacity of a network

**Figure 5:**
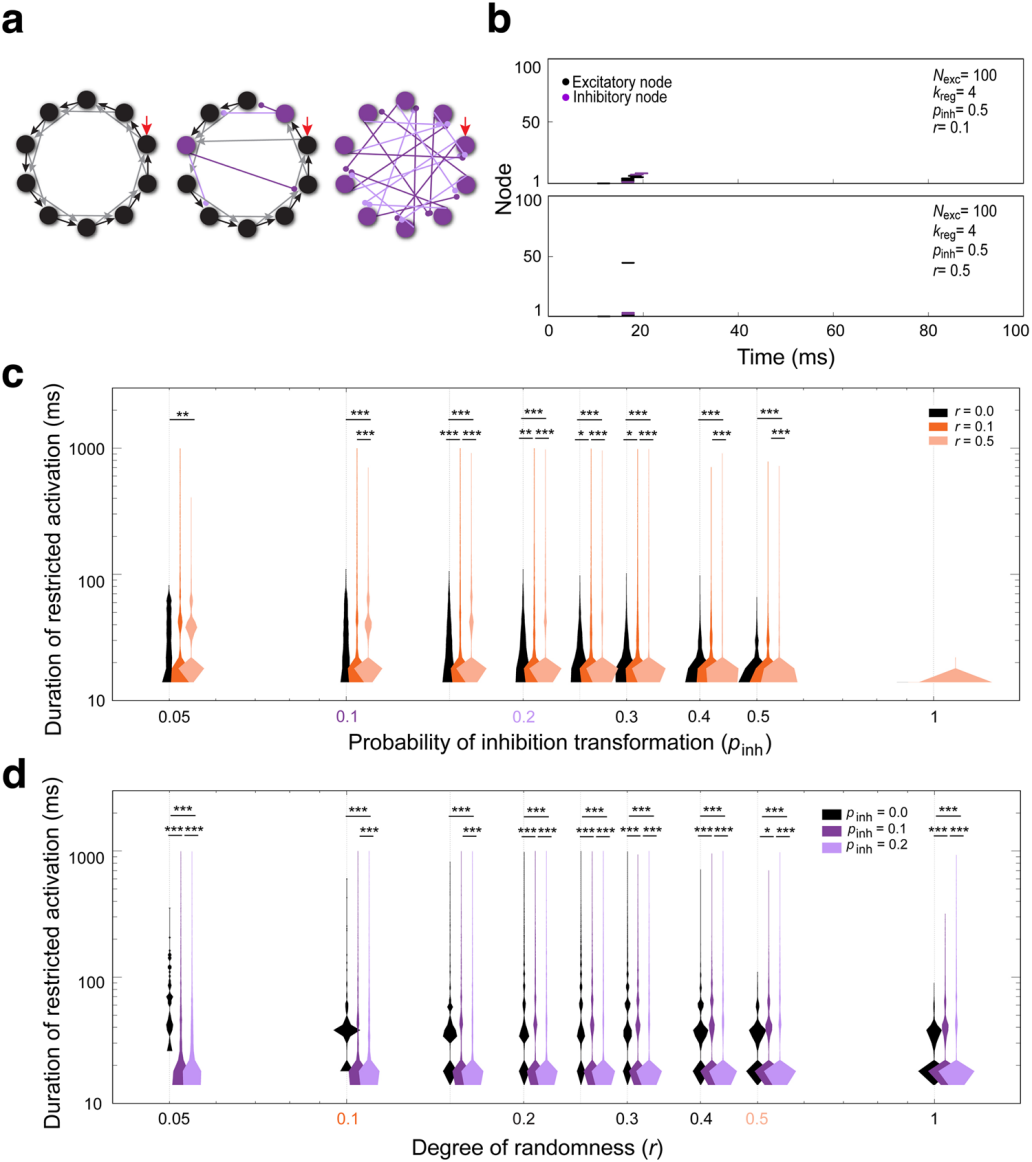
Random assignment of excitatory and inhibitory nodes combined with random rewiring of connections between nodes enhances the encoding capacity of a network. (**a**) Diagram depicting random assignment of excitatory and inhibitory nodes in the basic regular network with different degrees of randomness in network rewiring. *Left*: basic regular network structure, in which *p*_inh_ = 0 and *r* = 0. *Middle*: one edge of an excitatory node and an inhibitory node were rewired, respectively. As *p*_inh_ increases, increasing numbers of nodes are assigned as inhibitory. *Right*: when *p*_inh_ = 1 and *r* = 1, all nodes are inhibitory and all edges are rewired randomly. (**b**) The dynamics of network activation are shown as spike raster plots. When a node is transformed from excitatory to inhibitory, its color code is switched from black to magenta. Note that when *p*_inh_ = 0.5, *r* = 0.1, some simulated networks show ceased activation shortly after stimulation (*top*). Similar results were also observed in a network structure with *p*_inh_ = 0.5, *r* = 0.5 (*bottom*). (**c**) Excitatory nodes were randomly assigned to be transformed to inhibitory nodes in the basic regular network (*N*_exc_ = 100, *k*_reg_ = 4) at different degrees of randomness (*r* = 0 (black), 0.1 (orange) and 0.5 (light orange)). The distribution of restricted activation duration at a given *p*_inh_ is shown as a violin plot. Significance was assessed with Mann–Whitney *U*-test. Statistically significant differences are indicated; *p < 0.05, **p < 0.01, ***p < 0.001 (see Table S3). (**d**) Three distinct networks with *p*_inh_ = 0 (black), 0.1 (magenta), or 0.2 (light magenta) were examined. The distribution of restricted activation duration is shown as a violin plot. Significance was assessed with Mann–Whitney *U*-test. Statistically significant differences are shown; *p < 0.05, **p < 0.01, ***p < 0.001 (Table S3).

We next asked whether randomness could rescue the low encoding capacity caused by strong inhibition (Supplementary Fig. 1d). Introducing randomness to a network with a certain ratio of inhibitory nodes will increase the number of irregular network structures (Fig. 5a). Yet we found that in a network with high inhibition, introducing randomness had very little effect on the probability of activation restriction (e.g. *p*_inh_ = 0.3 to 1) (Supplementary Fig. S3c) or the duration of activation restriction (Fig. 5b). We therefore turned our attention to the effects of randomness on network activation durations under conditions of low inhibition.

We found that for simulated networks with different ratios of inhibitory nodes (e.g. *p*_inh_ = 0.05 to 0.15), both low degree and high degree of randomness (*r* = 0.1 and 0.5, respectively) effectively redistribute the activation durations into a multimodal distribution, suggesting that encoding reliability is enhanced without greatly sacrificing encoding capacity (Fig. 5c). However, such an effect of randomness is abolished when the inhibition is high (e.g. *p*_inh_ = 0.5 to 1, Fig. 5c).

To better understand how randomness enhances the encoding reliability of networks with low inhibition, we further examined the distribution of activation durations for networks with low inhibition and variable degrees of randomness. We found that under low inhibition, only a certain range of randomness (e.g. *r* = 0.2 to 0.5, *p*_inh_ = 0.1) caused the simulated network structures to exhibit a multimodal distribution of oscillation duration (Fig. 5d).

These results demonstrated that a certain degree of randomness effectively enhanced the encoding reliability of network structures. However, this only occurs in networks with low inhibition and not those with strong inhibition. While these results were informative, in that they demonstrated both randomness and inhibition can influence encoding capacity and reliability in regular circuits, the simulated networks did not explain why the fly olfactory LN network has such a high ratio of inhibitory LNs.

### Constructing an excitatory-inhibitory network by adding inhibitory nodes to a regular excitatory network

**Figure 6:**
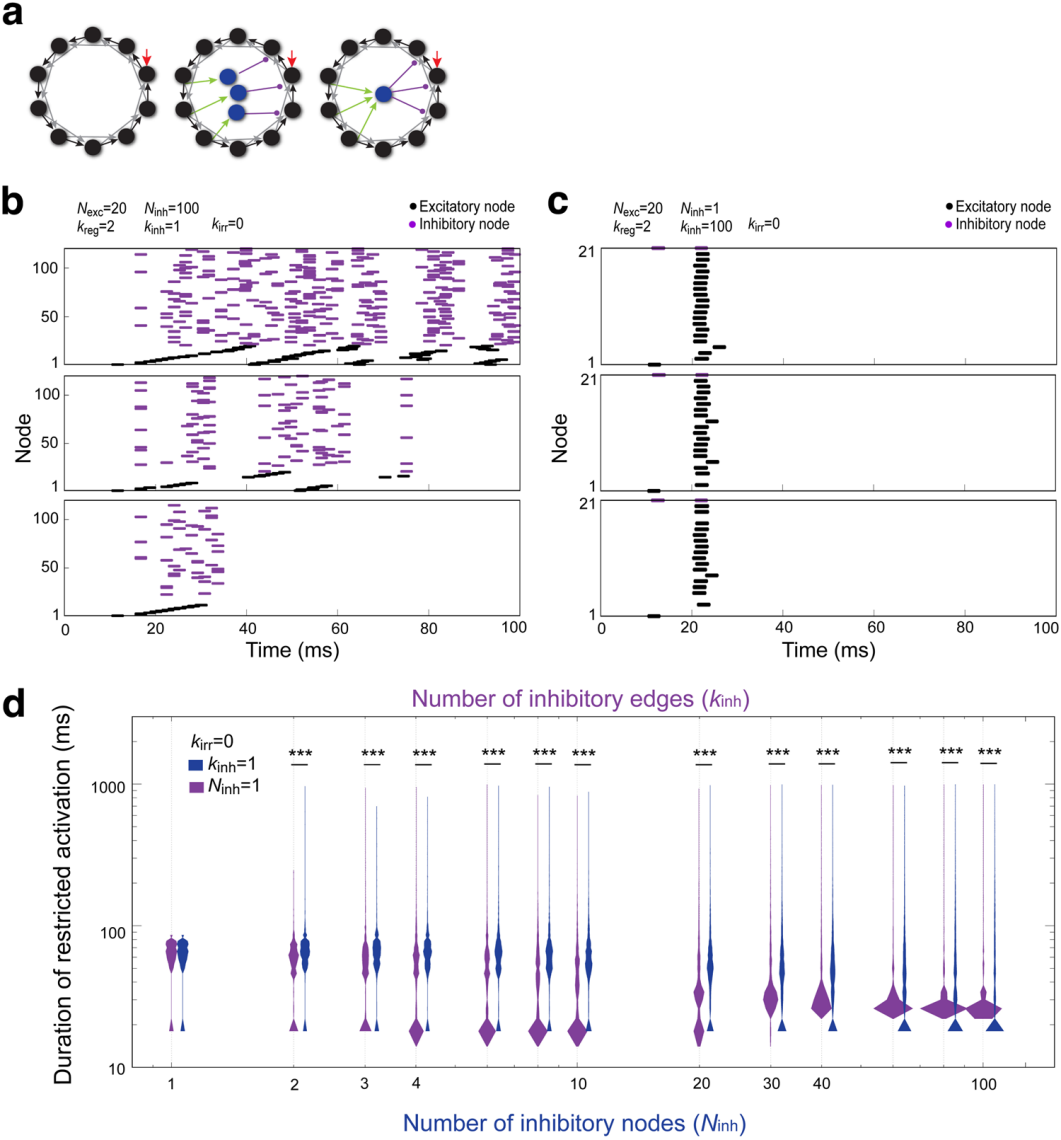
Building excitatory-inhibitory networks by adding inhibitory nodes and edges to simulate LN diversity. (**a**) *Left*: the basic regular network structure, in which the number of attached inhibitory nodes (*N*_inh_) and the number of attached inhibitory edges (*k*_inh_) are 0. Here, the number of irregular edges (*k*_irr_) of all simulated network structures is 0. *Middle*: three inhibitory nodes (blue, *N*_inh_ = 3) are added to the basic network structure, with each receiving a single excitatory supply edge (green) and extending a single inhibitory edge (magenta, *k*_inh_ = 1). *Right*: an additional inhibitory node (blue, *N*_inh_ = 1) is added to the basic network structure, along with three excitatory supply edges (green) and three inhibitory edges (magenta) at *k*_inh_ = 3. The number of additional excitatory supply edges and the number of inhibitory edges for each node was matched in all simulated networks. (**b**,**c**) The dynamics of network activation are shown as spike raster plots, when inhibition occurred at lower levels over time (**b**) or when strong inhibition was applied to the system at a single time (**c**). The three plots in each panel show representative data from samples with activation durations in the 75, 50 and 25-percentiles, as shown in the dark blue (**b**) and magenta (**c**) violin plots of (**d**). (**d**) The duration of restricted activation (∈ [15,1000]) from 5000 simulated network structures with different *N*_inh_ at a set value of *k*_inh_ = 1, or 5000 simulated network structures with different *k*_inh_ in a network with a set value of *N*_inh_ = 1 are shown as blue or magenta violin plots, respectively. Statistically significant differences were identified among groups of *k*_inh_ = 1 and among groups of *N*_inh_ = 1 by the Kruskal-Wallis *H*-test (*p* < 0.0001, Table S2). In addition, statistically significant differences between the two groups (e.g. *k*_inh_ = 1 and *N*_inh_ = 1) were found with the Mann–Whitney *U*-test. Only those comparisons with significant differences are indicated. *p < 0.05, **p < 0.01, ***p < 0.001 (Table S3).

Fly olfactory LNs are highly diverse in their morphologies^15^. According to the innervation patterns and AL coverage, these LNs can be categorized as pan-glomerular LNs (innervating whole AL), all-but-few glomerular LNs (innervating almost all glomeruli), regional LNs (innervating ~20 out of 54 continuous glomeruli), patchy LNs (innervating discontinuous glomeruli with patch-like neurite clusters), oligo-glomerular LNs (only innervating 3–5 glomeruli) and bilateral LNs (innervating both ALs)^15^. To simulate networks with distinct types of inhibitory LNs (Supplementary Fig. S1e), we first established a basic regular network (having 20 excitatory nodes, *k*_reg_ = 2) and added inhibitory nodes (*N*_inh_) that received input from one and relayed output to one excitatory node (*k*_inh_ = 1), simulating oligo-glomerular LNs (Fig. 6a, middle; mimicking the circuit shown in Supplementary Fig. S1e, middle). A key difference between this and the preceding strategy (Figs 3–5) is the number of excitatory edges to each inhibitory node and the number of inhibitory edges from the node differ, meaning that the strength of inhibition for a single node also differs. As the number of newly recruited inhibitory nodes reached 100, the simulated network (20 excitatory nodes and 100 inhibitory nodes) would have a similar ratio to the biological LN network.

Because many inhibitory LNs are pan-glomerular, all-but-few glomerular or regional LNs^15^, we also tested an excitatory-inhibitory network design wherein the network had only one recruited inhibitory node (*N*_inh_ = 1), but the number of inhibitory edges (*k*_inh_) from this node to other excitatory nodes was variable (Fig. 6a, right; mimicking the circuit shown in Supplementary Fig. S1e, bottom).

Conceptually, the number of total inhibitory edges equals *N*_inh_ × *k*_inh_ so both types of network should have the same number of total inhibitory edges when *N*_inh_ = *k*_inh_. Therefore, it was surprising that the networks did not exhibit similar efficiencies at restricting network activity oscillation (Fig. 6b,c, Supplementary Fig. S4a). The probable explanation for the different outcomes is that the timing of inhibitory inputs is quite different for the two networks. When *N*_inh_ is fixed at 1 (Fig. 6a, right), all inhibitory signals (through edges derived from *k*_inh_) are released simultaneously. In contrast, when *k*_inh_ is fixed at 1 (Fig. 6a, middle), the inhibitory nodes (based on *N*_inh_) release inhibitory signals individually, creating a unique temporal profile for each network structure. Therefore, when the inhibition properties of the simulated network approach experimentally determined values from biological LN networks, both networks in Figs 3a and 6a/right exhibit strong inhibition, while networks from Fig. 6a/middle exhibit weaker but prolonged inhibition over time.

We therefore asked if the different types of LNs have distinct effects on restricted activation durations. We found that in a network with solely oligo-glomerular LNs (*k*_inh_ = 1, *N*_inh_ varied; blue violin plots, Fig. 6d), adding more nodes to the network slightly prolonged the activation duration but broadened the distribution of activation durations. In networks simulating other types of oligo-glomerular LNs that innervate few glomeruli (*N*_inh_ = 1, *k*_inh_ = 1–10; magenta violin plots, Fig. 6d), the distribution of activation duration became bimodal with an increasing number of edges, suggesting that the encoding reliability was enhanced at the expense of encoding capacity. In networks simulating regional LNs (*N*_inh_ = 1, *k*_inh_ = 20–60; magenta violin plots, Fig. 6d), the distribution of activation duration changed from bimodal to unimodal with a relatively dense distribution of activation durations, suggesting the encoding reliability was enhanced with partial recovery of encoding capacity. When the network is composed of all-but-few glomerular LNs or pan-glomerular LNs (*N*_inh_ = 1, *k*_inh_ = 80–100; magenta violin plots in Fig. 6d), the encoding reliability is further enhanced. Therefore, the data suggest that distinct types of inhibitory LNs may have very different effects on network encoding capacity and encoding reliability.

### Attaching additional excitatory edges to the excitatory-inhibitory network to mimic circuit variability in interneuron network

**Figure 7:**
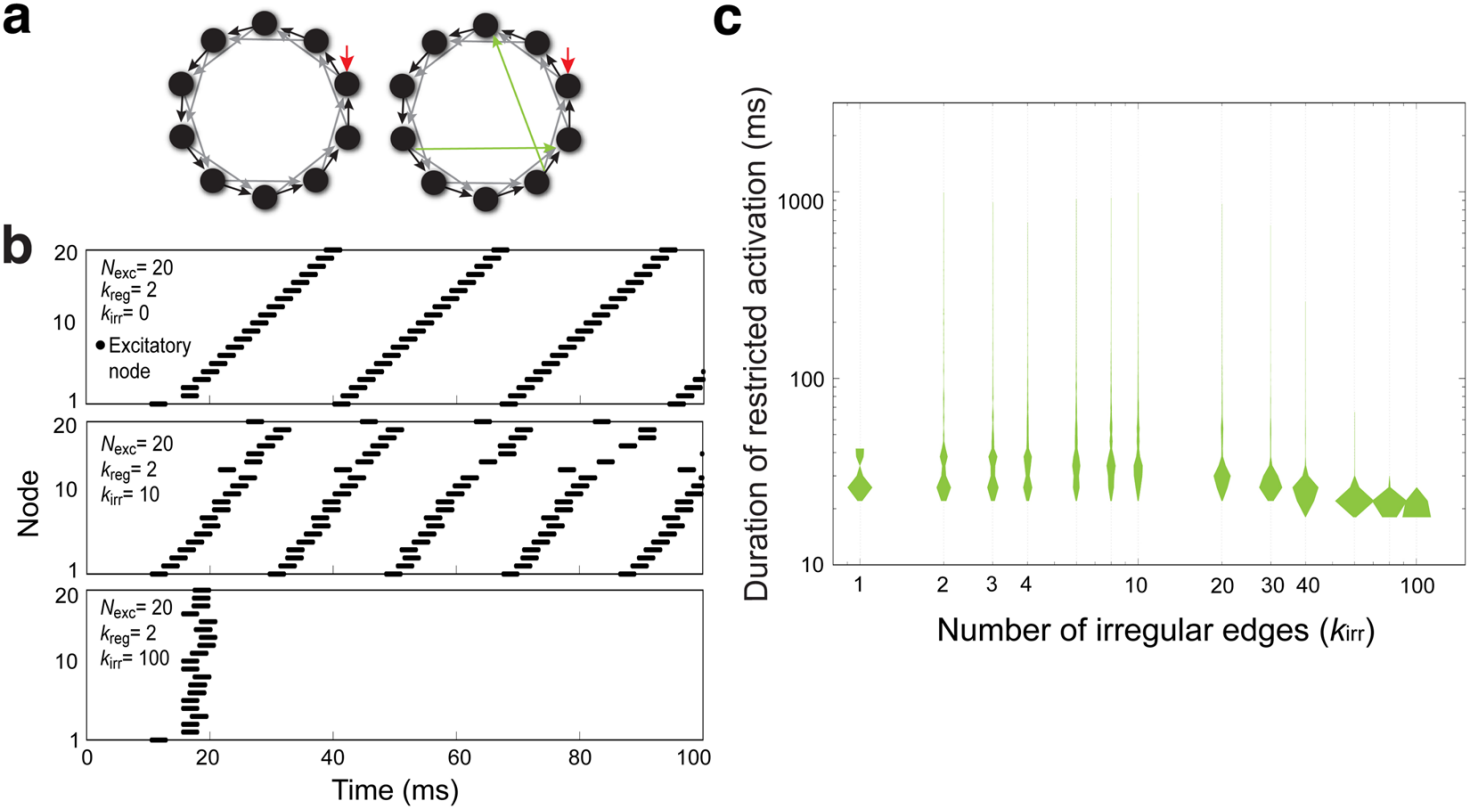
Attaching additional edges to the excitatory LN network to simulate LN variability. (**a**) Diagram depicting the random addition of edges to the basic regular network. *Left*: basic regular network, in which the number of irregular edges (*k*_irr_) is 0. *Right*: a network structure with two additional irregular edges (green, *k*_irr_ = 2). Two additional directed edges are randomly attached to two excitatory nodes, and randomly connected to two other nodes. (**b**) The dynamics of network activation are shown as spike raster plots. *Top*: When *k*_irr_ = 0, the network showed ceaseless and regular activation patterns. *Middle*: when *k*_irr_ = 10, some simulated networks show irregular and restricted activation patterns over time (example trace, 106.03 ms). *Bottom*: when *k*_irr_ = 100, the simulated networks showed irregular and restricted activation patterns with very short duration of activation (example trace, 20.96 ms). (**c**) The durations of restricted activation (green, ∈ [15,1000]) are shown as violin plots. When *k*_irr_ = 0, none of the simulated network structures exhibited restricted activation, and these data are not shown on the plot. A statistically significant difference was identified among groups by the Kruskal-Wallis *H*-test (*p* < 0.0001) (Table S2).

Circuit variability may originate from increased connections among existing LNs (Supplementary Fig. S1f). To simulate how additional excitatory connections contribute to the global activation of the network, we irregularly attached additional edges (*k*_irr_) to a basic regular network. Each additional excitatory directed edge bridged two randomly chosen excitatory nodes (Fig. 7a). The number of possible network structures can be as many as ${[\frac{2{N}_{{\rm{exc}}}!}{2!({N}_{{\rm{exc}}}-2)!}]}^{{k}_{{\rm{irr}}}}$. Accordingly, *N*_exc_(*N*_exc_ − 1) and [*N*_exc_(*N*_exc_ − 1)]^100^ possible network structures may be generated when *k*_irr_ = 1 and 100, respectively.

We first set *N*_exc_ = 20 and *k*_reg_ = 2 in the basic regular network, and then attached irregular edges. As expected, the oscillation patterns of network activity were more irregular when irregular edges were introduced to the network (Fig. 7b, middle). As the number of introduced irregular edges increased, some of the network structures exhibited activity restriction during the simulation period (Fig. 7b, bottom, Supplementary Fig. S4b). Notably, adding a sufficient number of irregular edges effectively prolonged the oscillation duration of network activation (increased encoding capacity) but this effect was at the expense of encoding reliability (2 ≥ *k*_irr_ ≥ 30, Fig. 7c). The network with *k*_irr_ = 10 seemed to represent the best condition in terms of showing balanced duration of restricted activation and probability of activation restriction. Therefore, this network was used for the subsequent experiments.

### Attaching additional excitatory edges expands the enhanced encoding capacity of a network with additional recruited inhibitory nodes

**Figure 8:**
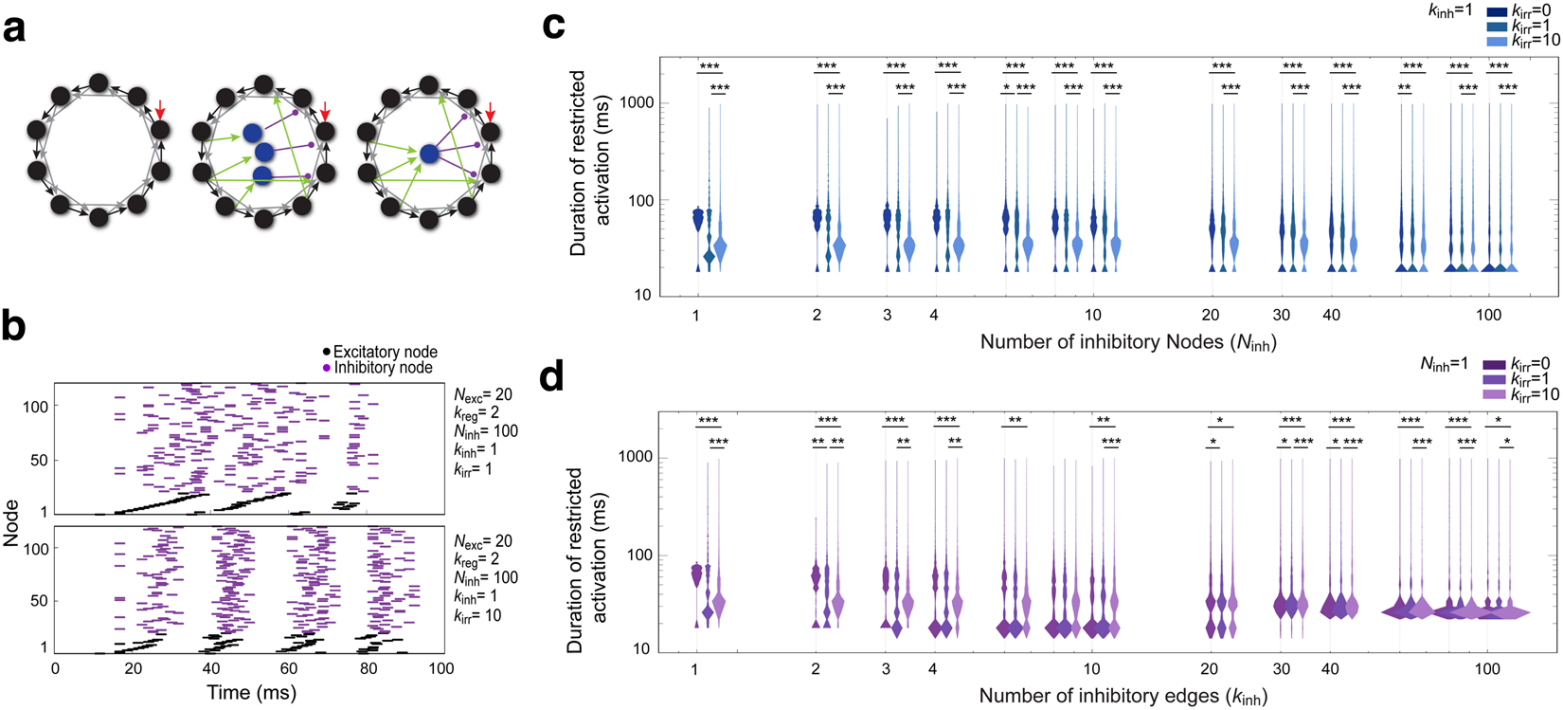
Attaching additional excitatory edges in combination with addition of inhibitory nodes expands the encoding capacity of a network. (**a**) Diagram depicting the random addition of excitatory edges and inhibitory nodes to the basic regular network. *Left*: the basic regular network structure, in which *k*_irr_ = 0. *Middle*: a network structure with two irregular edges (green, *k*_irr_ = 2), and three additional inhibitory nodes (blue, *N*_inh_ = 3). The irregular edges originated from existing excitatory nodes in the network and randomly extended to other excitatory nodes. Each added inhibitory node possesses one excitatory supply edge (green) and one inhibitory projection edge (magenta) at *k*_inh_ = 1. *Right*: a network structure with two irregular edges (green, *k*_irr_ = 2) and an additional inhibitory node (blue, *N*_inh_ = 1) with three excitatory supply edges (green) and three inhibitory projection edges (magenta, *k*_inh_ = 3). (**b**) The dynamics of network activation are shown as spike raster plots. (**c**) 5000 network structures were constructed by adding irregular edges (*k*_irr_ = 0, 1 or 10) to the basic regular network (*N*_exc_ = 20 and *k*_reg_ = 2) with a gradual increase in the number of inhibitory nodes (*N*_inh_ various, *k*_inh_ = 1). The distributions of restricted activation duration at a given number of inhibitory nodes are shown as violin plots. The violin plots are shown such that at a given *N*_inh_, the plot of *k*_irr_ = 0 is aligned over that *N*_inh_. (**d**) 5000 network structures were constructed by adding irregular edge (*k*_irr_) to the basic regular network with one additional inhibitory node that was associated with an increasing number of inhibitory edges (*N*_exc_ = 20, *N*_inh_ = 1, *k*_reg_ = 2, *k*_inh_ various). The violin plots at a given *k*_inh_ are shown such that the plot of *k*_irr_ = 0 is aligned over that *k*_inh_. Statistical significance was assessed with the Mann–Whitney *U*-test. *p < 0.05, **p < 0.01, ***p < 0.001 (Table S3).

In a complicated network system, such as the olfactory LN network, many sources of diversity and variation may simultaneously or sequentially influence a single stimulation event (Supplementary Fig. S1g). Therefore, we examined how multiple factors coordinately affect network activation oscillation. We randomly added excitatory edges (*k*_irr_) to a regular network with a gradual increase of either inhibitory nodes (*N*_inh_) (Fig. 8a, middle; mimicking Supplementary Fig. S1g, middle) or inhibitory edges (*k*_inh_) (Fig. 8a, right; mimicking Supplementary Fig. S1g, bottom). Not surprisingly, this cooperative effect dramatically concentrated the distribution of activation durations (enhanced the encoding reliability) at the expense of encoding capacity (Fig. 8c, *N*_inh_ = 1–10 and 8d, *k*_inh_ = 1–10). However, recruiting irregular edges mildly enhanced the activation duration of networks with regional LNs, all-but-few LNs and pan-glomerular LNs, with less effect on the distribution of activation duration (Fig. 8d, *k*_inh_ = 20–100). These results suggested that inhibitory LNs dominantly enhance the encoding capacity by expanding the network activation durations when the network wiring complexity is extremely high. In addition, circuit variability mildly enhances the encoding reliability of oligo-glomerular LNs or encoding capacity of regional LNs, all-but-few glomerular LNs and pan-glomerular LNs.

## Discussion

Neuronal diversity and variability are two fundamental features of neural circuits. Variations in network connections potentially confer greater complexity on a neural circuit and thus enhance the encoding capacity of the circuit. Such circuit variations, however, may also compromise the reliability of coding. Limited by current knowledge of neuronal types, numbers, and details of connections among neurons in a given circuit, it is far from clear how neuronal variability works with inhibition to influence encoding capacity and reliability of the circuit.

### Simulating LN network through ring model-like irregular structures

The diversity and variability of a biological neural network may result from morphological or biophysiological diversity. Although we have acquired substantial knowledge of the electrophysiological and morphological/anatomical characteristics of LNs at single cell resolution, we still do not fully understand the spatiotemporal firing dynamics of the LN ensemble upon odor stimulation. The goal of current work was to construct a model for studying the effects of diversity and inhibition on these dynamics. In order to do so, we simplified the model by excluding ORNs and PNs as a requisite precursor to future complex LN network simulations that will include ORNs and PNs. In fact, our model allows us to test both the diversity of connections and firing pattern diversity of individual neurons, because a given node will receive varying excitation and inhibition inputs along time.

We introduced different degrees of complexity to the simulated networks. One method we used was based on a small world model that allows edges in a ring structure to randomly connect to other nodes (Fig. 4). Under certain parameters of this model, one input stimulus may trigger ceaseless network activation. When the rewiring probability is progressively increased, the regularity of the ring structure decreases due to shortcuts in the network, resulting in a raised probability of ceased network activation^36,37^. Our results support such findings (Supplementary Figs S3, S4). Moreover, the delay time of the synaptic input also affects network activation and failure fractions^36,37^. It is not only structural changes that affect duration of network activation, as random networks with modifiable synapse strength also lead to membrane potential changes and irregular population spiking^38^.

In our simulated networks, we reason that as more distinct populations of nodes become involved in oscillation patterns, larger numbers of information objects can be encoded. In this way, the dynamics of oscillation patterns (activation duration) can be interpreted as reflecting the encoding capacity of a network. On the other hand, when simulated network structures, derived from a given network, exhibit highly similar durations of restricted activation, those network structures may have similar encoding capacity (Fig. 3d,e). Therefore, the distribution of restricted activation durations (irregularity of a network) may reflect encoding reliability.

### The diversity of inhibitory LNs likely contributes to optimized network encoding capacity, reliability and distinguishability

We employed two strategies to construct a diverse excitatory-inhibitory LN network. We found that a network constructed by adding 100 inhibitory nodes to a regular network of 20 excitatory nodes fits with the excitatory-inhibitory LN ratio of the *Drosophila* AL (Fig. 6). In such simulated networks, inhibition increased the activation duration (encoding capacity), in some cases at the expense of encoding reliability. However, in other instances, the encoding reliability was maintained or promoted, without sacrificing encoding capacity. This may explain why the biological LN network contains such a high ratio of inhibitory LNs.

We also simulated distinct types of inhibitory LNs and found they may differentially contribute to the encoding capacity and reliability of the network (Fig. 6). According to our results, regional inhibitory LNs function to enhance encoding capacity of networks, while slightly compromising encoding reliability (e.g. *N*_inh_ = 1, *k*_inh_ = 20–40, Fig. 6d) compared to oligo-glomerular LNs (e.g. *N*_inh_ = 1, *k*_inh_ = 5, Fig. 6d). However, all-but-few glomerular or pan-glomerular inhibitory LNs enhance the encoding reliability while compromising the encoding capacity (e.g. *N*_inh_ = 1, *k*_inh_ = 80–100, Fig. 6d). In a network containing oligo-glomerular LNs, the inhibitory LNs effectively enhanced the encoding capacity without greatly affecting encoding reliability (e.g. *N*_inh_ = 2 to 10, *k*_inh_ = 1, Fig. 6d). In the case that all inhibitory LNs are this type of neuron, the trade-off between encoding capacity and reliability becomes obvious (e.g. *N*_inh_ = 100, *k*_inh_ = 1, Fig. 6d).

Upon odor stimulation, distinct subsets of inhibitory and excitatory LNs are likely to be activated sequentially over a certain period of time. Thus the inhibition of biological LN networks may be dynamic, sometimes with low and other times with high inhibition. According to our simulations, if all inhibitory LNs were pan-glomerular, the LN network would usually exhibit very short network activation oscillation. On the other hand, if all inhibitory LNs were oligo-glomerular, the LN network would have a long but variable network activation duration, representing an extremely large encoding capacity but low encoding reliability. We propose that the high diversity of inhibitory LNs confers optimal encoding capacity, reliability and distinguishability through the interplay of temporal and spatial coding.

### LN variability may coordinate with inhibitory effects to enhance the encoding capacity and distinguishability of LN networks

We introduced circuit variability through different strategies (see Supplementary note). In our simulated networks, introducing variability to oligo-glomerular LNs compromised encoding reliability when the variability was low but enhanced encoding reliability when variability was moderate (Fig. 8c and *k*_inh_ = 1–10, Fig. 8d). On the other hand, introducing variability to networks with regional LNs, all-but-few glomerular LNs and pan-glomerular LNs mildly enhanced the encoding capacity without sacrificing encoding reliability (*k*_inh_ = 20–100, Fig. 8d). These results lead us to conclude that distinct types of inhibitory LNs mostly regulate encoding capacity, while circuit variability may enhance encoding reliability.

Our work helps to explain why the olfactory circuit contains distinct types of inhibitory LNs and provides insight into how LN variability may coordinate with inhibition to enhance the encoding reliability of a LN network. While we only simulated single types of inhibitory LNs, in future work, it will be interesting to introduce multiple types of LNs and/or simultaneously inject multiple stimuli into the same network to test how the combinations regulate encoding capacity and reliability of networks.

## Methods

### Neuron model

The original Hodgkin-Huxley model was used to describe a nerve cell as a resistor-capacitor circuit composed of resistors and a capacitor which reflect biophysical characteristics of ion channels and the cell membrane, respectively^34^. Considering that the cell membrane is a lipid bilayer structure, a capacitor *C*_*m*_ was introduced to describe the involvement of excitability. For the passage of ions through the cell membrane, voltage-dependent ion channels were considered as resistors. Accordingly, the membrane potential *V* of an excitatory neuron was defined as:

1 $${C}_{m}\frac{{\rm{d}}V}{{\rm{d}}t}=I-{g}_{{\rm{K}}}(V-{E}_{{\rm{K}}})-{g}_{{\rm{Na}}}(V-{E}_{{\rm{Na}}})-{g}_{{\rm{L}}}(V-{E}_{{\rm{L}}}).$$

*I* is an applied current. Terms on the right side of the equation represent potassium current (*g*_K_(*V* − *E*_K_)), sodium current (*g*_Na_(*V* − *E*_Na_)), and potential leak of channels (*g*_L_(*V* − *E*_L_)). *E*_K_, *E*_Na_, and *E*_L_ are reversal potentials of the corresponding channels. *g*_K_, *g*_Na_, and *g*_L_ are the conductance of corresponding channels, which were characterized as:

2 $$\begin{array}{rcl}{g}_{{\rm{K}}} & = & {\bar{g}}_{{\rm{K}}}{n}^{4}\\ {g}_{{\rm{Na}}} & = & {\bar{g}}_{{\rm{Na}}}{m}^{3}h\\ {g}_{{\rm{L}}} & = & {\bar{g}}_{{\rm{L}}}\end{array}$$

${\bar{g}}_{{\rm{K}}}$, ${\bar{g}}_{{\rm{Na}}}$, and ${\bar{g}}_{{\rm{L}}}$ are the maximum conductance of corresponding channels. *n*, *m*, and *h* were introduced as potential gates within [0, 1] that control the performance of *g*_K_, *g*_Na_, and *g*_L_ adapting to potential *V*, respectively. The factor *n*^4^ in *g*_*K*_ and the factor *m*^3^*h* in *g*_Na_ are related to the transport of ions through potassium and sodium channels, respectively. Ionic flows are restrained by:

3 $$\begin{array}{rcl}\frac{dm}{dt} & = & {\alpha }_{m}(1-m)-{\beta }_{m}m\\ \frac{dn}{dt} & = & {\alpha }_{n}(1-n)-{\beta }_{n}n\\ \frac{dh}{dt} & = & {\alpha }_{h}(1-h)-{\beta }_{h}h\end{array}$$

Here, *α*_*m*_, *β*_*m*_, *α*_*n*_, *β*_*n*_, *α*_*h*_, and *β*_*h*_ are voltage-dependent factors determining the potential gates, set as:

4 $$\begin{array}{ll}{\alpha }_{m}=\frac{0.1(V+25)}{\exp (\frac{V+25}{10})-1} & {\beta }_{m}=4\,\exp (V/18)\\ {\alpha }_{n}=\frac{0.01(V+10)}{\exp (\frac{V+10}{10})-1} & {\beta }_{n}=0.125\,\exp (V/80)\\ {\alpha }_{h}=0.07\,\exp (V/20) & {\beta }_{h}=\frac{1}{\exp (\frac{V+30}{10})-1}\end{array}$$

To extend the Hodgkin-Huxley model for individual neurons (nodes) to a modeled neuron that presumably receives multiple inputs in simulated neural circuits, we introduced a coupling term to each node to allow the integration of multiple inputs in this node. Consequently, the applied current in the *i*th neuron *I*_*i*_ was divided into two terms, one term *I*_*i*,sti_ being the stimulating current and the other term *I*_*i*,syn_ being the synaptic current. *I*_*i*,sti_ represents the current acquired from external stimuli. *I*_*i*,syn_ represents the current acquired from a set of pre-synapses *Q*_*i*_, which was defined as:

5 $${I}_{i,{\rm{syn}}}={\sum }_{j\in {Q}_{i}}{g}_{j,{\rm{syn}}}({V}_{j}-{V}_{{\rm{th}}})\,{\rm{\Phi }}({V}_{j}-{V}_{{\rm{th}}}).$$

Φ(*V*_*j*_ − *V*_th_) is a step function that equals 1 when *V*_*j*_ ≥ *V*_th_; otherwise Φ(*V*_*j*_ − *V*_th_) is 0. This allows *I*_*i*,syn_ to be set to follow the all-or-none phenomenon of action potentials that is observed in all characterized neurons^39^. Only when a physical quantity, such as the membrane potential or the current of a given neuron, is above the firing threshold (e.g. a constant potential threshold), the neuron fires and elicits an action potential. Here, *g*_*j*,syn_ and *V*_th_ are the conductance of inward currents from pre-synapses and the constant threshold potential of pre-synapses, respectively. The constant parameters were set as listed in Table S1. The parameters relating to biophysical characteristics, such as ${\bar{g}}_{K}$, ${\bar{g}}_{{\rm{Na}}}$, ${\bar{g}}_{L}$, *E*_*K*_, *E*_Na_ and *E*_*L*_, were adopted from the original Hodgkin–Huxley model^34^. As *I*_*i*,sti_ holds within a short period as a pulse and varies in different trials, the threshold potential *V*_th_ can be roughly observed by the function of potential *V*_*i*_ versus time. The value of *g*_*i*,syn_, which acts as coupling strength, is judged by two critria. First, each Hodgkin-Huxley type neuron should be able to be activated, which means that *g*_*i*,syn_ must be sufficiently large or the post-synaptic neuron needs to connect with multiple pre-synaptic neurons. Second, the Hodgkin-Huxley type model should avoid simulated crush, which means that *g*_*i*,syn_ cannot be set too large. If the value of *g*_*i*,syn_ falls into a range fitting within both criteria, our qualitative result would remain consistent.

### Input and network activation

All simulation work were performed with Cm = 1 μF/cm^2^, using CVODES with BDF and Newton iteration^40^. A sole 10 ms stimulus was injected to the simulated network structure 5 ms after the initiation of simulation. Because we anticipated that the stimulus would evoke an action potential for transmitting signals, the amplitude and duration of the stimulating current *I*_*i*,sti_ (and *τ*_*i*,sti_) were coordinately modulated. When *I*_*i*,sti_ and *τ*_*i*,sti_ were assigned as 2.5 μA/cm^2^ and 10 ms, respectively, and applied to a given node, the excited node would be depolarized following the integration of a series of inputs. The simulation time through this study was fixed at 1100 ms (constant sampling time ${\rm{\Delta }}t=0.01$ for Fig. 3d,e). When the global activation of a simulated network structure ceased during the simulation period, the network structure was considered to have undergone activation restriction. When an endless global activation happened to leave firing at the end of the simulation time (1100 ms), the program identifies it as a restricted activation. To avoid such a misidentification, we set a criterion (1000 ms) which was shorter than the simulation time to remove cases in which endless global activation ceased at time points at around 1100 ms. Accordingly, the duration of restricted activation was shorter than 1000 ms, but longer than the end time point of the stimulus (15 ms) used in this study.

### Euclidean distance and dot product analysis

We treated the collective state of nodes in a given network structure at each time step as a vector. Each vector had *n*-orthogonal coordinates in which the *i*th component indicated the state of the *i*th node. The distinguishability between two vectors $\vec{a}$ and $\vec{b}$ were partially quantified by either the Euclidean distance $|\vec{a}-\vec{b}|$ or the inner product $\vec{a}\cdot \vec{b}$. One limitation of Euclidean distance is that the value of Euclidean distance does not identify distinguishability if $|\vec{a}|$ and $|\vec{b}|$ are too small. On the other hand, the inner product is limited if $\vec{a}=c\vec{b}$, in which *c* is a constant. The normalized Euclidean distance was calculated as $|\vec{a}-\vec{b}|/(|\vec{a}|+|\vec{b}|)$. When the angle between two vectors is 180°, the corresponding normalized Euclidean distance equals the sum of the length of two vectors. The results of inner product analysis were normalized as $\vec{a}\cdot \vec{b}/|\vec{a}||\vec{b}|$.

### Script used in this study

The script used in this study will be available from the open database generated by the corresponding author laboratory.

### Immunostaining of local interneurons

The four *GAL4* insertions, *GH298*, *H24*, *NP3056*, and *OK107*, collectively labeled almost all local interneurons in the AL^15^. Adult brains of flies carrying these four *GAL4* lines were dissected and immunostained with antibodies as previously described^15^. The primary antibodies used were: rat anti-mCD8 Ab (1:100, Invitrogen MCD0800), rabbit anti-GABA Abs (1:200, Sigma A2052) and rabbit anti-VGlut Abs (1:1000, a gift from Dr. Aaron DiAntonio). Secondary antibodies were goat anti-rat IgG-Alexa Flour® 488 (Invitrogen, A11006), goat anti-rabbit IgG-Cy^TM^3 (Jackson ImmunoResearch Laboratory; 111-165-144), and Zenon^TM^ Alexa Fluor^TM^ 647 Rabbit IgG Labeling Kit (Invitrogen, Z25308). Since both anti-GABA and anti-VGlut antibodies were raised from rabbits, Zenon^TM^ Alexa Fluor^TM^ 647 Rabbit IgG Labeling Kit was used to label rabbit anti-GABA antibodies and goat anti-rabbit IgG-Cy^TM^3 was used to label rabbit anti-VGlut antibodies, respectively. Images were collected using a LSM510 confocal microscope (Carl Zeiss). The number of glutamatergic LNs (but not that of GABAergic nor of both) from this experiment was previously published^15^.

### Data availability

The parameters used in this study can be found in Table S1 of the Supplementary Information. The scripts and instructions used to run the simulations in this paper will be available from an open database generated by the corresponding author laboratory or upon request.

## Electronic supplementary material

Supplementary Information
