# Supplementary material for: Circuit variability interacts with excitatory-inhibitory diversity of interneurons to regulate network encoding capacity
Source: Sci Rep. 2018 May 23;8:8027. doi: 10.1038/s41598-018-26286-8 (PMC5966413; doi:10.1038/s41598-018-26286-8)
Supplement: Supplementary file 1 — Supplementary Information [file 41598_2018_26286_MOESM1_ESM.pdf]

Supplementary Information

Circuit variability interacts with excitatory-inhibitory diversity of interneurons to regulate network encoding capacity

Kuo-Ting Tsai, Chin-Kun Hu, Kuan-Wei Li, Wen-Liang Hwang, Ya-Hui Chou*

Supplementary Note 1. Probability of activation restriction

When the network activity ceased during the simulation period, the network structure was categorized as having undergone activation restriction. The probability of activation restriction is defined as the percentage of network structures exhibiting restricted activation in a total of 5000 tested network structures. For instance, when the probability of activation restriction is 0.4, 40% of 5000 simulated network structures exhibited cessation of network activation after oscillation.

Supplementary Note 2. Introducing circuit variability to the simulated networks

We introduced circuit variability through different strategies. For instance, the circuit variability of networks in Figure 5 was produced by rewiring the connections of existing nodes to introduce randomness (r) or by transforming excitatory edges to inhibitory edges (p_{inh}). In the networks shown in Figure 6, the circuit variability was introduced by recruiting additional N_{inh} (as a consequence, the total number of nodes in the network increased) and the random assignment of nodes that received inhibitory edges from those newly recruited inhibitory nodes. Networks derived from these two strategies have different origins of circuit variability and the strength of introduced network inhibitions over time is different. Interestingly, the first type of circuit variability enhances the network encoding capacity only when the inhibition is low (Figure 5). The effect of the second type of circuit variability is mainly on enhancing the encoding reliability via randomly assignment of nodes receiving inhibitor edges from newly added nodes or enhancing the encoding capacity via recruiting additional inhibitory nodes of networks (Figure 6).

Supplementary Table 1. Parameters used in this study.

Parameter	Definition	Value
C_m	Membrane capacity	1 μF/cm^2
$\bar{g}_{\mathrm{K}}$	Maximum conductance for K^+	36 mS/cm^2
$\bar{g}_{\mathrm{Na}}$	Maximum conductance for Na^+	120 mS/cm^2
$\bar{g}_{\mathrm{L}}$	Conductance for other ions	0.3 mS/cm^2
E_{K}	Equilibrium potential for K^+	-12 mV
E_{Na}	Equilibrium potential for Na^+	115 mV
E_{L}	Equilibrium potential for other ions	10.6 mV
$I_{i,\mathrm{sti}}$	Stimulating current	2.5 μA/cm^2
$\tau_{i,\mathrm{sti}}$	Duration of stimulating current	10 ms
V_{th}	Threshold potential	20 mV
$g_{j,\mathrm{syn}}$	Conductance of inward currents from pre-synapses	0.077 mS/cm^2, if node j is excitatory<br>-0.077 mS/cm^2, if node j is inhibitory

Supplementary Table 2. Statistical analyses results for Figures 3c, 4c, 6d, and 7c.

Figure	p-Value
Fig. 3c	< 2.2e-16
Fig. 4c	< 2.2e-16
Fig. 6d (k_{inh}=1)	< 2.2e-16
Fig. 6d (N_{inh}=1)	< 2.2e-16
Fig. 7c	< 2.2e-16

* Kruskal-Wallis H test was used.

Supplementary Table 3. Statistical analyses results for Figures 5c, 5d, 6d, 8c and 8e.

Figure	Condition	Pairs	p-Value
Fig. 5c	p_{inh} =0.05	r =0 vs r =0.1	0.5586
		r =0 vs r =0.5	0.001447
		r =0.1 vs r =0.5	0.3925
	p_{inh} =0.1	r =0 vs r =0.1	0.2806
		r =0 vs r =0.5	3.38e-11
		r =0.1 vs r =0.5	2.67e-12
	p_{inh} =0.15	r =0 vs r =0.1	3.01e-09
		r =0 vs r =0.5	9.42e-05
		r =0.1 vs r =0.5	< 2.2e-16
	p_{inh} =0.2	r =0 vs r =0.1	0.001005
		r =0 vs r =0.5	2.97e-6
		r =0.1 vs r =0.5	3.26e-13
	p_{inh} =0.25	r =0 vs r =0.1	0.02233
		r =0 vs r =0.5	6.61e-09
		r =0.1 vs r =0.5	5.82e-10
	p_{inh} =0.3	r =0 vs r =0.1	0.04505
		r =0 vs r =0.5	2.89e-13
		r =0.1 vs r =0.5	4.79e-11
	p_{inh} =0.35	r =0 vs r =0.1	0.8416
		r =0 vs r =0.5	4.87e-12
		r =0.1 vs r =0.5	3.69e-07
	p_{inh} =0.5	r =0 vs r =0.1	0.5567
		r =0 vs r =0.5	7.75e-10
		r =0.1 vs r =0.5	5.51e-07
	p_{inh} =1	r =0 vs r =0.1	1
		r =0 vs r =0.5	0.3174
		r =0.1 vs r =0.5	0.3174
Fig. 5d	r =0.05	p_{inh}=0 vs p_{inh}=0.1	6.78e-07
		p_{inh}=0 vs p_{inh}=0.2	2.67e-13
		p_{inh}=0.1 vs p_{inh}=0.2	1.03e-11
	r =0.1	p_{inh}=0 vs p_{inh}=0.1	7.13e-02
		p_{inh}=0 vs p_{inh}=0.2	1.19e-05
		p_{inh}=0.1 vs p_{inh}=0.2	1.01e-07
	r =0.15	p_{inh}=0 vs p_{inh}=0.1	0.9971
		p_{inh}=0 vs p_{inh}=0.2	3.69e-14
		p_{inh}=0.1 vs p_{inh}=0.2	< 2.2e-16
	r =0.2	p_{inh}=0 vs p_{inh}=0.1	3.08e-13
		p_{inh}=0 vs p_{inh}=0.2	< 2.2e-16
		p_{inh}=0.1 vs p_{inh}=0.2	< 2.2e-16
	r =0.25	p_{inh}=0 vs p_{inh}=0.1	< 2.2e-16
		p_{inh}=0 vs p_{inh}=0.2	< 2.2e-16
		p_{inh}=0.1 vs p_{inh}=0.2	< 2.2e-16

	r =0.3	p_{inh}=0 vs p_{inh}=0.1	< 2.2e-16
		p_{inh}=0 vs p_{inh}=0.2	< 2.2e-16
		p_{inh}=0.1 vs p_{inh}=0.2	< 2.2e-16
	r =0.35	p_{inh}=0 vs p_{inh}=0.1	6.58e-14
		p_{inh}=0 vs p_{inh}=0.2	< 2.2e-16
		p_{inh}=0.1 vs p_{inh}=0.2	< 2.2e-16
	r =0.5	p_{inh}=0 vs p_{inh}=0.1	0.02013
		p_{inh}=0 vs p_{inh}=0.2	< 2.2e-16
		p_{inh}=0.1 vs p_{inh}=0.2	6.55e-13
	r =1	p_{inh}=0 vs p_{inh}=0.1	1.90e-05
		p_{inh}=0 vs p_{inh}=0.2	< 2.2e-16
		p_{inh}=0.1 vs p_{inh}=0.2	< 2.2e-16
Fig. 6d	k_{irr} =0	k_{inh}=1, N_{inh}=1 vs N_{inh}=1, k_{inh}=1	1
		k_{inh}=1, N_{inh}=2 vs N_{inh}=1, k_{inh}=2	1.71e-12
		k_{inh}=1, N_{inh}=3 vs N_{inh}=1, k_{inh}=3	< 2.2e-16
		k_{inh}=1, N_{inh}=4 vs N_{inh}=1, k_{inh}=4	< 2.2e-16
		k_{inh}=1, N_{inh}=6 vs N_{inh}=1, k_{inh}=6	< 2.2e-16
		k_{inh}=1, N_{inh}=8 vs N_{inh}=1, k_{inh}=8	< 2.2e-16
		k_{inh}=1, N_{inh}=10 vs N_{inh}=1, k_{inh}=10	< 2.2e-16
		k_{inh}=1, N_{inh}=20 vs N_{inh}=1, k_{inh}=20	< 2.2e-16
		k_{inh}=1, N_{inh}=30 vs N_{inh}=1, k_{inh}=30	< 2.2e-16
		k_{inh}=1, N_{inh}=40 vs N_{inh}=1, k_{inh}=40	< 2.2e-16
		k_{inh}=1, N_{inh}=60 vs N_{inh}=1, k_{inh}=60	< 2.2e-16
		k_{inh}=1, N_{inh}=80 vs N_{inh}=1, k_{inh}=80	< 2.2e-16
		k_{inh}=1, N_{inh}=100 vs N_{inh}=1, k_{inh}=100	< 2.2e-16
Fig. 8c	k_{inh}=1, N_{inh}=1	k_{irr}=0 vs k_{irr}=1	7.72e-01
		k_{irr}=0 vs k_{irr}=10	< 2.2e-16
		k_{irr}=1 vs k_{irr}=10	7.25e-10
	k_{inh}=1, N_{inh}=2	k_{irr}=0 vs k_{irr}=1	6.79e-01
		k_{irr}=0 vs k_{irr}=10	< 2.2e-16
		k_{irr}=1 vs k_{irr}=10	< 2.2e-16
	k_{inh}=1, N_{inh}=3	k_{irr}=1 vs k_{irr}=10	5.53e-01
		k_{irr}=0 vs k_{irr}=10	< 2.2e-16
		k_{irr}=1 vs k_{irr}=10	< 2.2e-16
	k_{inh}=1, N_{inh}=4	k_{irr}=0 vs k_{irr}=1	3.88e-01
		k_{irr}=0 vs k_{irr}=10	< 2.2e-16
		k_{irr}=1 vs k_{irr}=10	< 2.2e-16
	k_{inh}=1, N_{inh}=6	k_{irr}=0 vs k_{irr}=1	4.89e-02
		k_{irr}=0 vs k_{irr}=10	< 2.2e-16
		k_{irr}=1 vs k_{irr}=10	< 2.2e-16
	k_{inh}=1, N_{inh}=8	k_{irr}=0 vs k_{irr}=1	5.65e-01
		k_{irr}=0 vs k_{irr}=10	< 2.2e-16
		k_{irr}=1 vs k_{irr}=10	< 2.2e-16
	k_{inh}=1, N_{inh}=10	k_{irr}=0 vs k_{irr}=1	1.99e-01
		k_{irr}=0 vs k_{irr}=10	< 2.2e-16
		k_{irr}=1 vs k_{irr}=10	< 2.2e-16
	k_{inh}=1, N_{inh}=20	k_{irr}=0 vs k_{irr}=1	1.59e-01

		k_{irr}=0 vs k_{irr}=10	< 2.2e-16
		k_{irr}=1 vs k_{irr}=10	< 2.2e-16
	k_{inh}=1, N_{inh}=30	k_{irr}=0 vs k_{irr}=1	7.63e-02
		k_{irr}=0 vs k_{irr}=10	< 2.2e-16
		k_{irr}=1 vs k_{irr}=10	8.74e-14
	k_{inh}=1, N_{inh}=40	k_{irr}=0 vs k_{irr}=1	9.58e-01
		k_{irr}=0 vs k_{irr}=10	8.86e-09
		k_{irr}=1 vs k_{irr}=10	1.15e-07
	k_{inh}=1, N_{inh}=60	k_{irr}=0 vs k_{irr}=1	1.13e-02
		k_{irr}=0 vs k_{irr}=10	0.000361
		k_{irr}=1 vs k_{irr}=10	0.3645
	k_{inh}=1, N_{inh}=80	k_{irr}=0 vs k_{irr}=1	2.60e-01
		k_{irr}=0 vs k_{irr}=10	1.50e-10
		k_{irr}=1 vs k_{irr}=10	1.03e-07
	k_{inh}=1, N_{inh}=100	k_{irr}=0 vs k_{irr}=1	8.04e-01
		k_{irr}=0 vs k_{irr}=10	4.36e-15
		k_{irr}=1 vs k_{irr}=10	1.76e-14
Fig. 8d	k_{inh}=1, N_{inh}=1	k_{irr}=0 vs k_{irr}=1	7.72e-01
		k_{irr}=0 vs k_{irr}=10	< 2.2e-16
		k_{irr}=1 vs k_{irr}=10	7.25e-10
	k_{inh}=2, N_{inh}=1	k_{irr}=0 vs k_{irr}=1	8.89e-03
		k_{irr}=0 vs k_{irr}=10	< 2.2e-16
		k_{irr}=1 vs k_{irr}=10	2.11e-03
	k_{inh}=3, N_{inh}=1	k_{irr}=0 vs k_{irr}=1	5.70e-02
		k_{irr}=0 vs k_{irr}=10	1.52e-13
		k_{irr}=1 vs k_{irr}=10	4.36e-03
	k_{inh}=4, N_{inh}=1	k_{irr}=0 vs k_{irr}=1	2.24e-01
		k_{irr}=0 vs k_{irr}=10	1.01e-08
		k_{irr}=1 vs k_{irr}=10	1.51e-03
	k_{inh}=6, N_{inh}=1	k_{irr}=0 vs k_{irr}=1	1.47e-01
		k_{irr}=0 vs k_{irr}=10	4.31e-03
		k_{irr}=1 vs k_{irr}=10	6.68e-01
	k_{inh}=8, N_{inh}=1	k_{irr}=0 vs k_{irr}=1	6.83e-01
		k_{irr}=0 vs k_{irr}=10	5.00e-01
		k_{irr}=1 vs k_{irr}=10	1.49e-01
	k_{inh}=10, N_{inh}=1	k_{irr}=0 vs k_{irr}=1	2.52e-01
		k_{irr}=0 vs k_{irr}=10	4.06e-03
		k_{irr}=1 vs k_{irr}=10	1.35e-05
	k_{inh}=20, N_{inh}=1	k_{irr}=0 vs k_{irr}=1	3.80e-02
		k_{irr}=0 vs k_{irr}=10	2.29e-02
		k_{irr}=1 vs k_{irr}=10	9.58e-01
	k_{inh}=30, N_{inh}=1	k_{irr}=0 vs k_{irr}=1	1.24e-02
		k_{irr}=0 vs k_{irr}=10	< 2.2e-16
		k_{irr}=1 vs k_{irr}=10	3.79e-15
	k_{inh}=40, N_{inh}=1	k_{irr}=0 vs k_{irr}=1	2.49e-02
		k_{irr}=0 vs k_{irr}=10	< 2.2e-16

	k_{irr}=1 vs k_{irr}=10	< 2.2e-16
k_{inh}=60, N_{inh}=1	k_{irr}=0 vs k_{irr}=1	7.29e-02
	k_{irr}=0 vs k_{irr}=10	< 2.2e-16
	k_{irr}=1 vs k_{irr}=10	< 2.2e-16
k_{inh}=80, N_{inh}=1	k_{irr}=0 vs k_{irr}=1	8.40e-01
	k_{irr}=0 vs k_{irr}=10	5.77e-06
	k_{irr}=1 vs k_{irr}=10	1.55e-05
k_{inh}=100, N_{inh}=1	k_{irr}=0 vs k_{irr}=1	7.68e-01
	k_{irr}=0 vs k_{irr}=10	2.06e-02
	k_{irr}=1 vs k_{irr}=10	3.79e-02

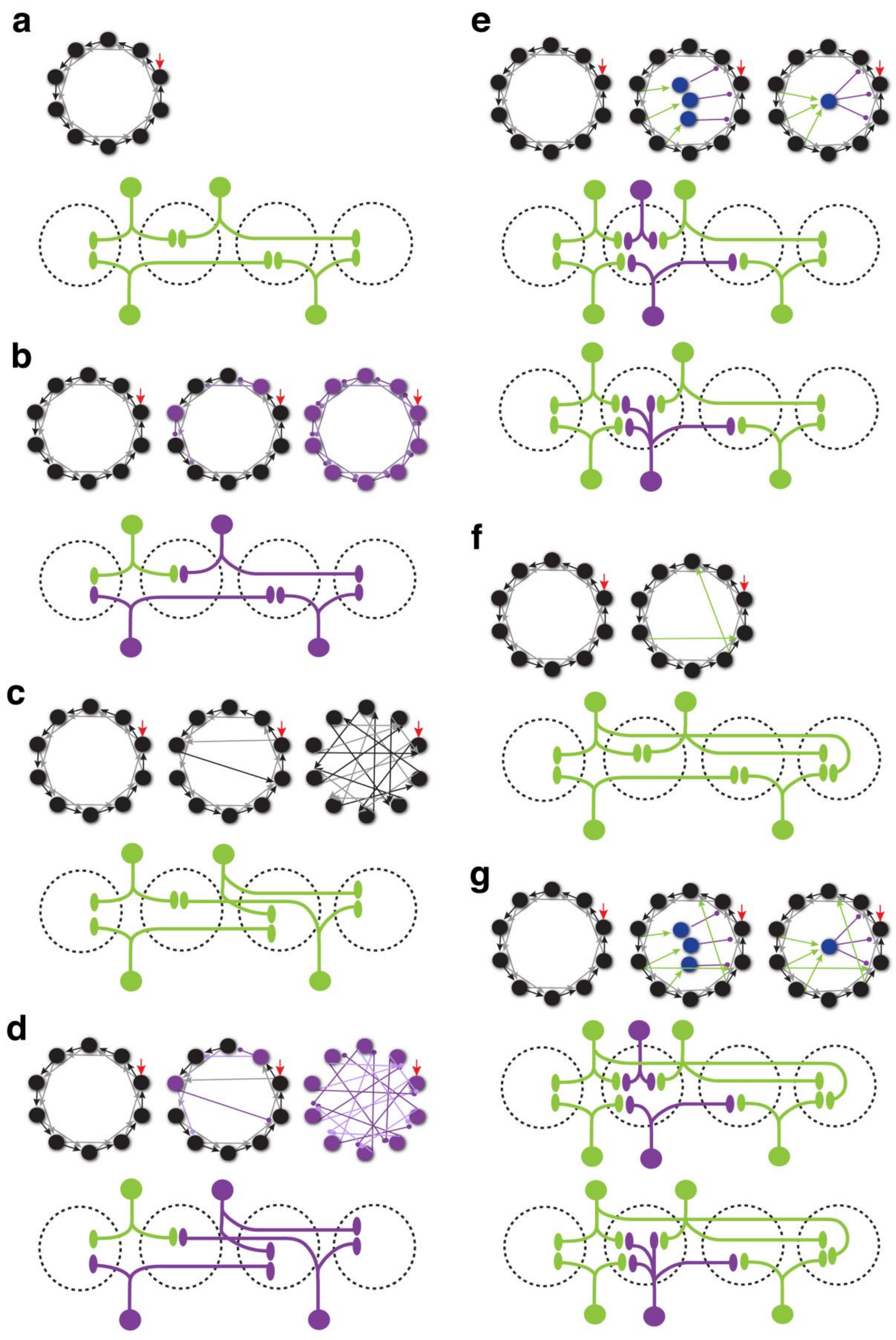
a
b
c
d
e
f
g

Supplementary Figure 1. Diagram of distinct types of closed networks and corresponding schematics of isolated LN networks. *Top*, diagrams of distinct closed networks. The diagrams of closed networks shown in (**a-g**) were reproduced from Figures 2b, 3a, 4a, 5a, 6a, 7a and 8a. *Bottom*, schematics of distinct isolated LN networks. (**a**) An isolated LN network (without ORNs or PNs) containing only excitatory LNs. (**b**) An isolated LN network with excitatory and inhibitory LNs. (**c**) An isolated LN network with solely excitatory LNs that exhibit variability in their connections (compared to A). (**d**) An isolated LN network with diverse excitatory and inhibitory LNs, and morphological variability in LN connections. (**e**) Isolated LN networks with excitatory and inhibitory LNs. *Middle*: network contains multiple inhibitory LNs with fewer synaptic connections. *Bottom*: network contains a single inhibitory LN with more synaptic connections. (**f**) Isolated excitatory LN network with additional connections between nodes. (**g**) An isolated LN network with diversity of excitatory and inhibitory LNs, and variability of LN connections. *Middle*: a network with multiple inhibitory LNs containing fewer synaptic connections as well as additional connections from excitatory LNs. *Bottom*: a network with a single inhibitory LN containing many more synaptic connections as well as additional connections from excitatory LNs.

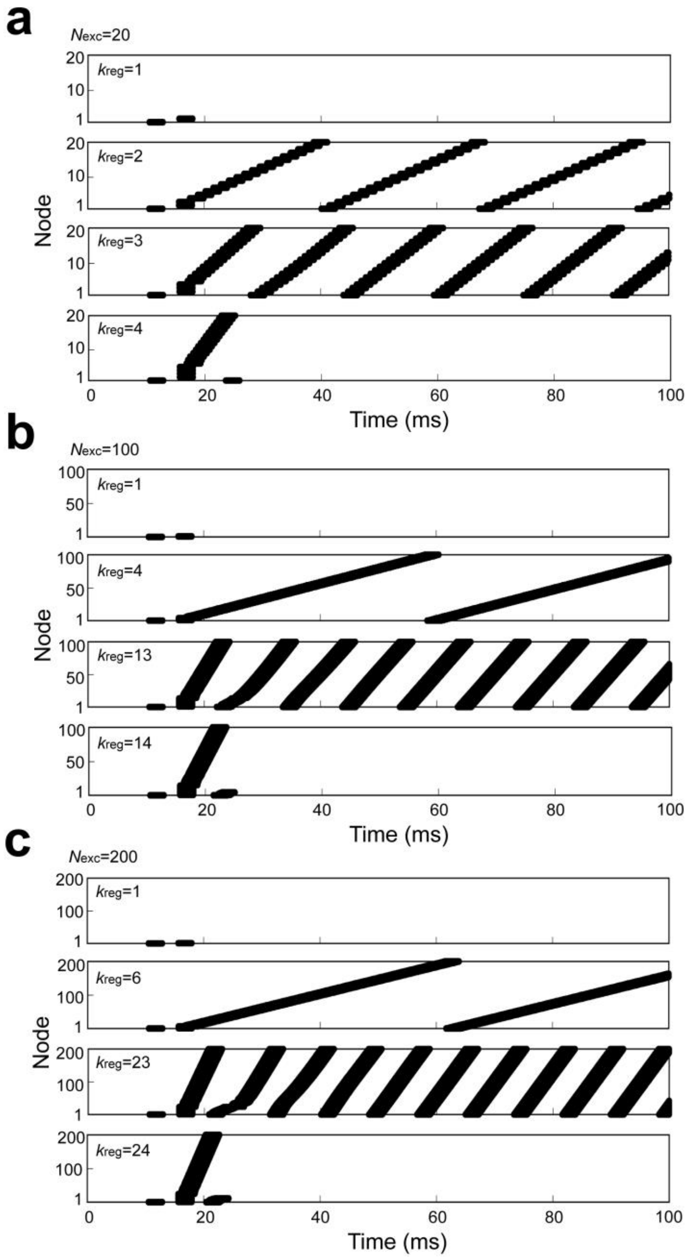

Supplementary Figure 2. The activation dynamics of regular circular networks with different numbers of nodes and edges. Various numbers of regular activation edges (e.g., k_{reg}=1, 2, 3, or 4 in the case of N_{exc}=20) were introduced to the network with nodes (N_{exc}) at 20 (**a**), 100 (k_{reg}=1, 4, 13, or 14) (**b**), or 200 (k_{reg}=1, 6, 23, or 24) (**c**). The dynamics of network activation are shown as spike raster plots, with the vertical axis denoting the identity of each node in the network (the node receiving injected stimulus is assigned as node 1) and the horizontal axis denoting simulation time (ms). The results in (**c**) were reproduced from Figure 2c.

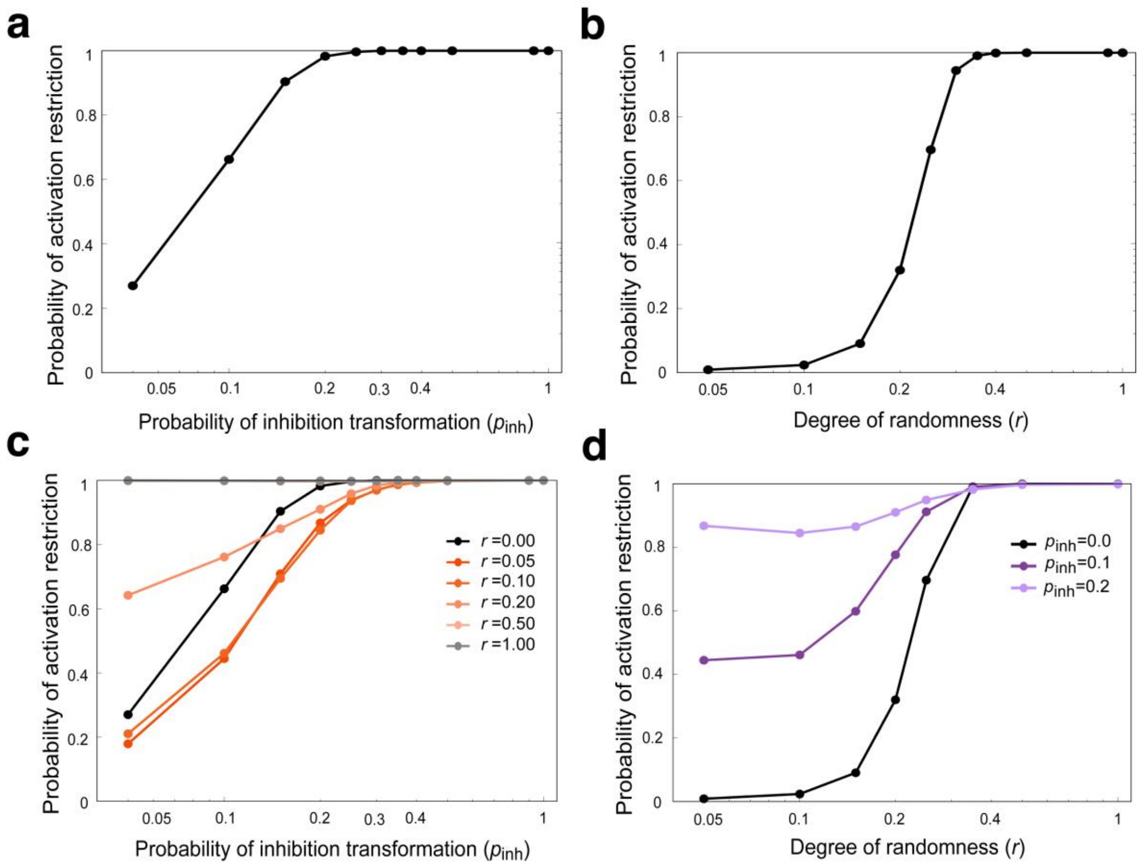

Supplementary Figure 3. The probability of activation restriction of simulated networks derived from strategy 1. (**a**) Five thousand networks ($N_{exc} = 100$, $k_{reg} = 4$) were simulated by randomly switching nodes from excitatory to inhibitory at a given probability of inhibitory transformation (p_{inh}). The probability of activation restriction (black dots and line, ∈[0,1]) represents the proportion of network structures with restricted activation. (**b**) Five thousand network structures were simulated by randomly rewiring the directed edges from nodes in the basic regular network ($N_{exc} = 100$, $k_{reg} = 4$) at a given randomness (r). The probability of activation restriction is shown as black dots and line (∈[0,1]). (**c**) Excitatory nodes were randomly assigned to inhibitory nodes in the basic regular network ($N_{exc} = 100$, $k_{reg} = 4$) at different degrees of randomness ($r = 0$, 0.05, 0.1, 0.2, 0.5, or 1). Networks with $r = 1$ (gray) performed similar to networks with $r = 0.5$ (light beige). Consequently, the plotted line and symbols for $r = 0.5$ are obscured by $r = 1$ data and are not clearly visible in the figure. (**d**) The inhibitory nodes in the networks were assigned with $p_{inh} = 0$ (black), 0.1 (magenta), or 0.2 (light magenta).

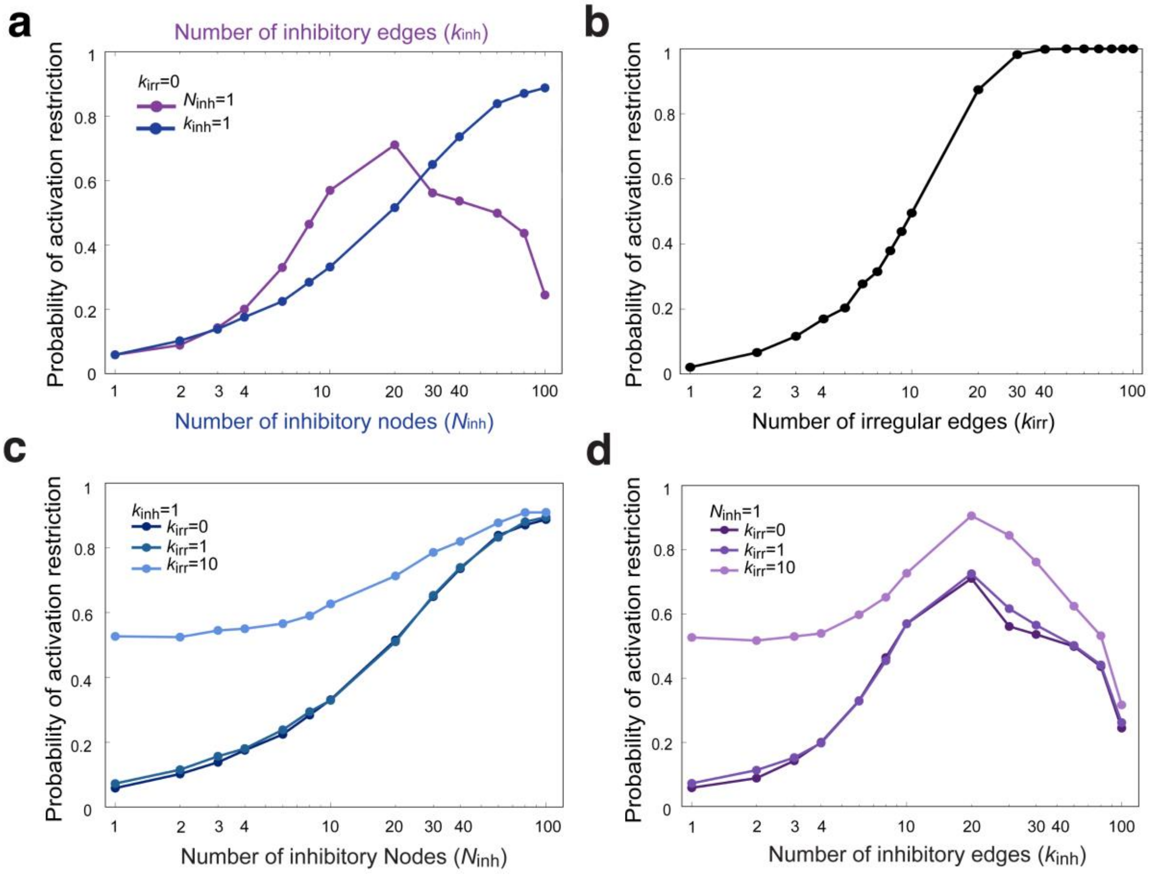

Supplementary Figure 4. The probability of activation restriction of simulated networks derived from strategy 2. (**a**) Network structures (5000 simulated network structures for each N_{inh}) were constructed by adding a given N_{inh} to the basic regular network ($N_{\mathrm{exc}} = 20$ and $k_{\mathrm{reg}} = 2$) with one additional inhibitory edge ($k_{\mathrm{inh}} = 1$) attached to each additional node (blue dots and line). Similarly, 5000 network structures were constructed by adding a given k_{inh} to the basic regular network ($N_{\mathrm{exc}} = 20$ and $k_{\mathrm{reg}} = 2$) with a single additional inhibitory node ($N_{\mathrm{inh}} = 1$, magenta dots and line). (**b**) Network structures (5000 structures for each k_{irr}) were constructed by attaching additional activation edges to nodes within the basic regular structure ($N_{\mathrm{exc}} = 20$ and $k_{\mathrm{reg}} = 2$). Higher k_{irr} indicates more excitatory edges are attached existing nodes. The probability of activation restriction (black dots and line, ∈[0,1]) represents the proportion of structures with restricted activation among 5000 simulated network structures. (**c**) Five thousand network structures were constructed by adding irregular edges ($k_{\mathrm{irr}} = 0$, 1 or 10) to the basic regular network ($N_{\mathrm{exc}} = 20$ and $k_{\mathrm{reg}} = 2$) with a gradual increase in the number of inhibitory nodes (N_{inh} various, $k_{\mathrm{inh}} = 1$). (**d**) Similar to (**c**), 5000 network structures were constructed by adding irregular edges (k_{irr}) to the basic regular network via one additional inhibitory node that had a variable number of inhibitory edges ($N_{\mathrm{exc}} = 20$, $N_{\mathrm{inh}} = 1$, $k_{\mathrm{reg}} = 2$, k_{inh} various).
